# Mediated nuclear import and export of TAZ and the underlying molecular requirements

**DOI:** 10.1038/s41467-018-07450-0

**Published:** 2018-11-23

**Authors:** Michael Kofler, Pam Speight, Darby Little, Caterina Di Ciano-Oliveira, Katalin Szászi, András Kapus

**Affiliations:** 1 Keenan Research Centre for Biomedical Science of the St. Michael’s Hospital, University of Toronto, Toronto, ON M5B 1T8 Canada; 2 Department of Surgery, University of Toronto, Toronto, ON M5B 1T8 Canada; 3 Department of Biochemistry, University of Toronto, Toronto, ON M5B 1T8 Canada

## Abstract

Nucleocytoplasmic distribution of Yap/TAZ is regulated by the Hippo pathway and the cytoskeleton. While interactions with cytosolic and nuclear “retention factors” (14–3–3 and TEAD) are known to control their localization, fundamental aspects of Yap/TAZ shuttling remain undefined. It is unclear if translocation occurs only by passive diffusion or via mediated transport, and neither the potential nuclear localization and efflux signals (NLS, NES) nor their putative regulation have been identified. Here we show that TAZ cycling is a mediated process and identify the underlying NLS and NES. The C-terminal NLS, representing a new class of import motifs, is necessary and sufficient for efficient nuclear uptake via a RAN-independent mechanism. RhoA activity directly stimulates this import. The NES lies within the TEAD-binding domain and can be masked by TEAD, thereby preventing efflux. Thus, we describe a RhoA-regulated NLS, a TEAD-regulated NES and propose an improved model of nucleocytoplasmic TAZ shuttling beyond "retention".

## Abstract (web summary)

The transcriptional co-factors Yap and TAZ are regulated by Hippo signalling and mechanical forces via their nucleocytoplasmic shuttling. Here the authors identify a RhoA-regulated C-terminal nuclear localization signal and a TEAD-regulated N-terminal nuclear export signal of TAZ in an epithelial cell line.

## Introduction

Yap and its paralog TAZ are transcriptional co-activators that play key roles in tissue growth, differentiation, development, and regeneration^1,2^. Moreover, dysregulation of Yap/TAZ emerges as an important pathogenic factor in cancer, arteriosclerosis, and—as recent studies including our own indicate—organ fibrosis^3–11^. Yap/TAZ are primarily controlled at the level of their nuclear accumulation (nucleocytoplasmic shuttling), which is affected by myriads of chemical and mechanical cues, including the integrity of cell–cell contacts (cell density), matrix stiffness, cellular tension, metabolic state, and soluble mediators^12–19^. Most of these inputs converge on two distinct, yet interdependent signal-transducing systems: the Hippo pathway and the state of the actomyosin cytoskeleton. The former regulatory mode is better understood; sequential activity of the Hippo core kinases MST1/2 and LATS1/2 keeps Yap/TAZ phosphorylated, which promotes their binding to “cytosolic retention factors”, primarily to 14–3–3 proteins^20,21^. Upon Hippo inactivation, Yap/TAZ translocate to the nucleus where they associate with various transcription factors, predominantly with members of the TEAD family, which also act as their “nuclear retention factors”^22–25^. The mechanisms underlying cytoskeleton-dependent localization are incompletely elucidated, but certain details are emerging; RhoA activation and actin polymerization have been shown to promote (de)binding from “retention factors” (e.g., AMOT)^26–28^, and a very recent work revealed that mechanical forces could increase the permeability of the nuclear pore^29^ thereby facilitating the nuclear accumulation of Yap.

However, fundamental questions about the nuclear shuttling of Yap/TAZ remain open. The majority of current views explain localization in terms of “retention models” wherein the critical determinants are the interactions of Yap/TAZ with binding partners. Given that many “retention factors” (e.g., 14–3–3 and TEAD) also shuttle^30,31^, these models alone may not adequately account for the compartmentalization of Yap/TAZ, a view also raised by a work published while our paper was under revision^32^. Importantly, none of the current models define the actual translocation processes themselves, which ensue once the molecules are “free”/transport-competent. Thus, it is uncertain if nuclear entry occurs via simple diffusion or if it is a mediated process. Supposing the latter, the structural requirements are unknown: neither nuclear localization sequence(s) (NLS) nor nuclear export sequence(s) (NES) have been identified. Further, it remains largely unclear if the translocation steps (nuclear entry and egress) per se are regulated. We addressed these essential questions by generating diffusion-limited TAZ constructs, inducible (rapamycin-triggered) influx and efflux systems, and by applying mutagenesis. Using these approaches we have identified a non-conventional NLS, necessary and sufficient for efficient import, which is regulated by RhoA. We also found a critical nuclear export signal (NES), which can be masked by TEAD.

## Results

### TAZ is imported through a facilitated process into the nucleus

**Fig. 1:**
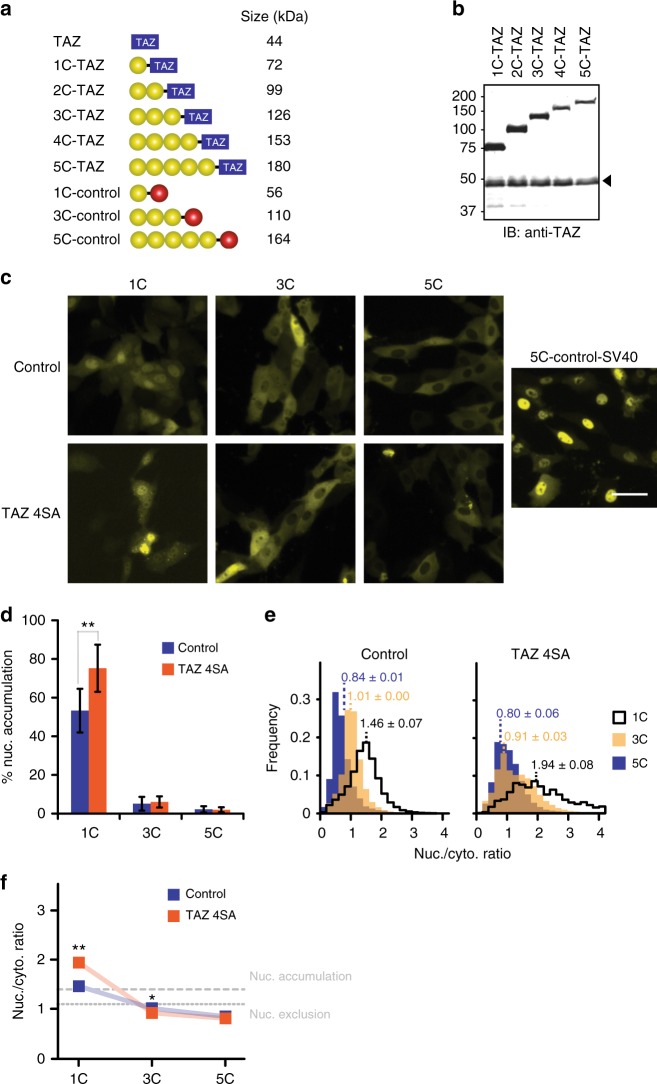
Large tags suppress nuclear accumulation of TAZ under steady-state conditions. **a** TAZ and a control (mCherry, red circle) were fused to increasing numbers of mCitrine molecules (yellow circles). Molecular weights are indicated. **b** Western blot analysis of transiently over-expressed TAZ constructs in LLC-PK1 cells, using a TAZ-specific antibody. Endogenous TAZ is marked by an arrowhead. An uncropped version is shown in Supplementary Figure 1A. **c** Fluorescence microscopy images of LLC-PK1 cells transiently expressing control or TAZ 4SA constructs with varying tag-sizes (numbers of mCitrine units). 5C-control-SV40 additionally comprises residues 114–135 of simian virus 40 (SV40) large tumor antigen that mediate strong nuclear import. This construct demonstrates that the 5C-tag does not abolish mediated import and serves as positive control for nuclear localization. Images depict mCitrine fluorescence. Scale bar represents 50 μm. Representative images of one experiment. Number of repeats: *n* = 3. **d** Visual quantification of cells with nuclear enrichment of control and TAZ 4SA constructs. Cells were scored as “nuclear” (nuc.) if nuclear fluorescence was visibly higher than cytoplasmic fluorescence. For each measurement, at least 100 cells were analysed. See also Supplementary Figure 3B. Data are represented as bar diagram, *n* = 7. Throughout this work, data from visual analyses are depicted in the same way. **e** Automated analysis of the localization of indicated constructs in cells, shown in (**c**), using the ImageXpress platform. For each construct, nucleocytoplasmic fluorescence ratios (*R*_N/C_) from more than 100 cells were obtained from the inbuilt automated cell-recognition algorithm and the medians of these ratio distributions were calculated. Depicted histograms are averages, *n* = 3. **f** Comparison of median *R*_N/C_, determined in (**e**). Dotted and dashed lines represent *R*_N/C_ thresholds for the classification of nuclear exclusion and accumulation, respectively, as described in Supplementary Figure 3C. In the following, all automated analyses are presented as scatter charts. Data are represented as means ± SD. *p < 0.05, **p < 0.01; Student’s *t*-test

**Fig. 2:**
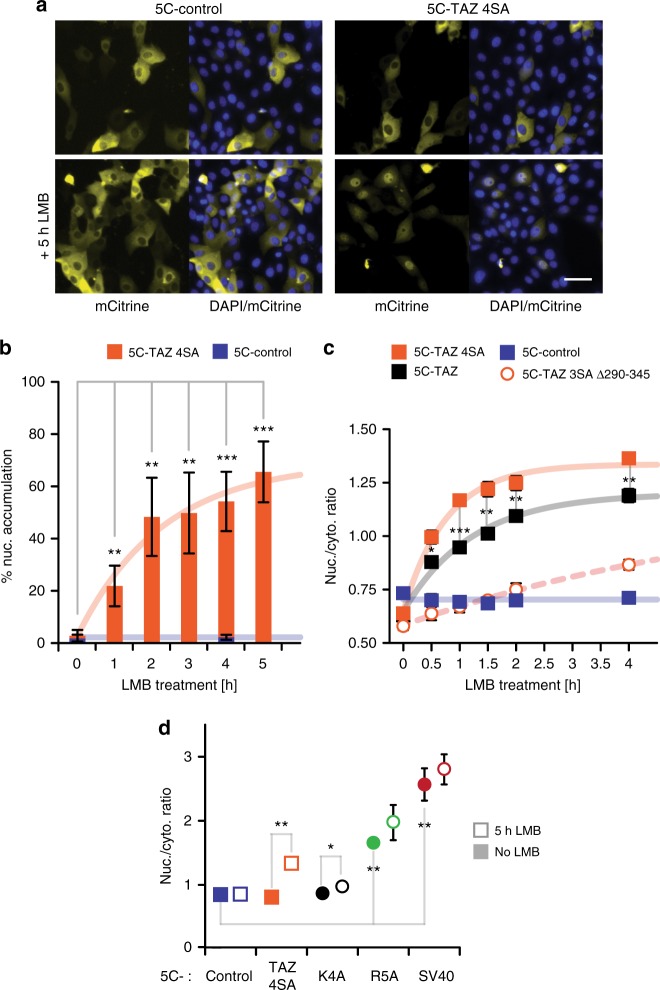
Nuclear import of TAZ is a mediated process observable upon LMB treatment. **a** LMB dependent nuclear accumulation of 5C-TAZ 4SA. Fluorescence microscopy images of cells expressing 5C-control or 5C-TAZ 4SA, without and with 5 h LMB treatment. 5C-control localizes predominantly to the cytoplasm, independent of LMB treatment, consistent with the absence of passive influx and mediated import. 5C-control, therefore, represents the negative control for nuclear localization. 5C-TAZ 4SA accumulates in the nucleus upon LMB treatment, suggesting the presence of a mediated import (and a LMB-sensitive export). Images depict mCitrine fluorescence (yellow) and a merge of mCitrine and DAPI (blue) channels. Scale bar represents 50 μm. **b** Visual quantification of nuclear accumulation. Cells expressing 5C-control or 5C-TAZ 4SA were incubated with LMB for the indicated times and nuclear accumulation was assessed by visual analysis, *n* = 4, 5, or 9 for individual constructs. Curve represents a fit to an exponential rise to maximum function. For the control, no time-dependence was observed and the line depicts the overall average accumulation. **c** Automated quantification of nuclear accumulation. Cells expressing 5C-control, 5C-TAZ 4SA, 5C-TAZ, or a deletion construct (5C-TAZ 3SA ∆290–345, introduced in the following sections), were treated with LMB for the indicated times and median *R*_N/C_ values were determined, *n* = 3 or 4 for individual constructs. Curves and line as described in (**b**). Differences between full-length TAZ constructs (5C-TAZ 4SA, 5C-TAZ) and 5C-control and differences between 5C-TAZ 4SA and the deletion construct (5C-TAZ 3SA ∆290–345) are significant at all time-points (*p* < 0.01, not indicated). Asterisks indicate significant differences between 5C-TAZ 4SA and 5C-TAZ. **d** Median *R*_N/C_ of 5C-TAZ 4SA, 5C-control, and the 5C-tag fused to the minimal SV40-NLS (residues 126–132) or its weaker variants (K4A, R5A), without and with 5 h LMB treatment, *n* = 3. Data are represented as means ± SD. *p < 0.05, **p < 0.01, ***p < 0.001; Student’s *t*-test

TAZ lacks any recognizable NLS that could account for a mediated import into the nucleus upon Hippo inactivation. To test whether nuclear translocation of TAZ involves facilitated transport, we studied its localization under circumstances when passive diffusion through the nuclear pore was minimized. Passive import can be reduced by artificially increasing the molecular weight with tags^33–35^. We based our tag-design on mCitrine building blocks since this fluorophore is inert to facilitated import^36^ and does not oligomerize^37^, which allows size-adjustments in increments of 27 kD. Constructed tags comprised one to five mCitrine molecules (1C- to 5C-tags) and gradually pushed the molecular weight of TAZ fusions beyond any reported exclusion limit for passive diffusion through the nuclear pore complex (NPC)^33–35^ (Fig. 1a, b). To ensure that LATS-dependent cytosolic “sequestration” by 14–3–3 proteins was not the limiting factor for the nuclear accumulation of TAZ fusions, we mutated all LATS-phosphorylation sites (TAZ 4SA), which was shown to drive TAZ into the nucleus^20,21^. In parallel, we fused the tags to a similar-sized control protein (mCherry) that distributes evenly throughout the cell^38^ to detect residual passive influx. The 1C-tag did not obstruct nuclear accumulation of TAZ 4SA, while 1C-control diffused freely, following the nuclear distribution of mCitrine alone^35,36^ (Fig. 1c, Supplementary Figure 2). 3C- and 5C-tags led to the exclusion of both TAZ 4SA and control from the nucleus. However, the large tags did not abolish mediated nuclear import per se, as seen with a construct comprising the strong NLS of simian virus 40 large T antigen (SV40; Fig. 1c, last panel). We quantified nuclear enrichment of these constructs by visual scoring (Fig. 1d, Supplementary Figure 3A and 3B) and by an automated approach that measured nuclear-to-cytoplasmic fluorescence ratios (R_N/C_; Fig. 1e, f, Supplementary Figure 3A and 3C). These analysis strategies compared well (Supplementary Figure 3D) and revealed higher nuclear accumulation of 1C-TAZ 4SA relative to 1C-control (Fig. 1d–f). Moreover, they confirmed a robust nuclear exclusion of the 5C-constructs. Importantly, the observed exclusion by no means proved the absence of facilitated import; an alternative explanation was that concomitant export outweighed influx. To distinguish the two possibilities, we inhibited the major nuclear export factor CRM1 with Leptomycin B (LMB) and thereby trapped shuttling proteins in the nucleus^39–41^. Upon LMB treatment, 5C-control remained cytoplasmic, whereas 5C-TAZ 4SA accumulated in the nucleus (Fig. 2a–c). This result established that TAZ 4SA, but not the control, shuttled between the two compartments through mediated import and LMB-sensitive export.

The displayed import activity of TAZ 4SA was substantially weaker than that of the strong SV40-NLS and ranked between the weak SV40 K4A mutant and the medium-strength R5A mutant^36^(Fig. 2d). This observation is consistent with the intricate regulation of TAZ localization (as opposed to constitutive nuclear accumulation of SV40^42^). In line with this notion, mediated nuclear uptake of TAZ is limited by LATS activity, since 5C-tagged wild-type (WT) TAZ accumulated less in the nucleus than 5C-TAZ 4SA when cells were treated with LMB (Fig. 2c). To delineate the mechanistic underpinning of mediated import, we sought to define the underlying structural requirements i.e., the TAZ region(s) necessary for import.

### Region 290–345 is sufficient and necessary for efficient TAZ nuclear localization

**Fig. 3:**
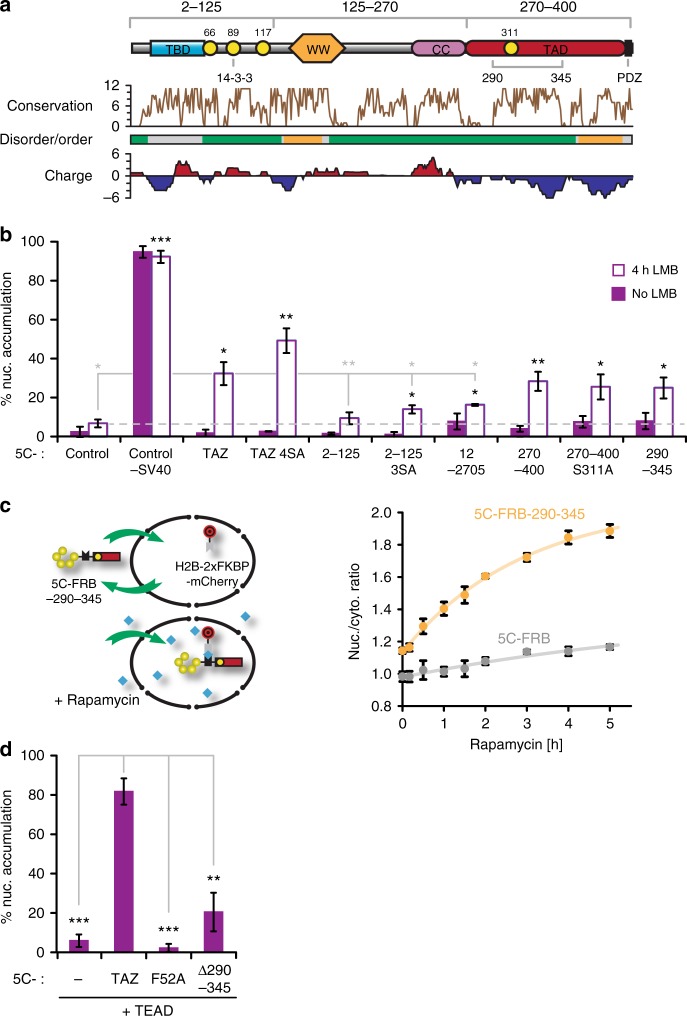
Identification of the TAZ-NLS. **a** Domain architecture of TAZ and TAZ fragments used in (**b**). LATS-phosphorylation sites S66, S89, S117, and S311 are shown as yellow circles. The sequence conservation plot was calculated using Jalview^78^. Disordered (green) and ordered (orange) regions were calculated with SPOT-Disorder^79^ and Metadisorder^80^. For regions in gray, predictions were not consistent. The charge plot represents local charges, averaged over a 19-residue window. Positive values are in red, negative values in blue. CC: coiled-coil region; PDZ: PDZ domain binding site; TAD: transactivation domain; TBD: TEAD-binding domain; WW: WW domain; 14–3–3: 14–3–3 binding site. **b** Nuclear enrichment of 5C-TAZ fragments without and with 4h LMB treatment, *n* = 3. Black and gray asterisks indicate significantly higher and lower nuclear accumulations in the presence of LMB, relative to control and 5C-TAZ, respectively. **c** RIS’N model showing rapamycin-induced nuclear entrapment of 5C-FRB-290–345 by H2B-2xFKBP-mCherry. Right: Median *R*_N/C_ values for 5C-FRB-290–245 (orange) and 5C-FRB (gray, control) were plotted against time of rapamycin treatment, *n* = 3, 4, or 7 for various time points. Statistically significant differences between the two probes were observed at all time-points (*p* < 0.01, not indicated). Curves are fits to an exponential rise to maximum function. **d** TEAD-mediated nuclear entrapment of WT and mutant 5C-TAZ constructs, *n* = 3. Data are represented as means ± SD. *p < 0.05, **p < 0.01, ***p < 0.001; Student’s *t*-test

We first analysed the localization of 5C-tagged TAZ fragments (Fig. 3a) in the absence and presence of LMB. Consistent with our results for full-length TAZ, all fragments tested were mostly cytoplasmic without treatment (Fig. 3b). In the presence of LMB most  tested fragments showed higher nuclear accumulation than 5C-control. Yet, only the C-terminal fragment (270–400) mediated import of similar magnitude as the full-length TAZ, and this accumulation was independent of the comprised LATS-phosphorylation site S311 (270–400 S311A). Thus, we concentrated on the C-terminal 131 aminoacids and further localized this import capacity to the highly conserved core region 290–345. Correspondingly, deletion of this region in 5C-TAZ 4SA mitigated the LMB-induced nuclear accumulation (Fig. 2c). Since LMB broadly inhibits efflux and alters the entire nuclear proteome^43^, we sought to study nuclear import under conditions that only blocked TAZ efflux in a highly specific and inducible manner. Therefore, we developed a system based on rapamycin-inducible sequestration in the nucleus, which we termed RIS’N. In this system, strong rapamycin-dependent heterodimerization between an immobile, nuclear FKBP domain and a 5C-tagged FKBP−rapamycin-binding domain (5C-FRB)^44^ traps shuttling 5C-FRB fusions in the nucleus (Fig. 3c, left panel). When co-expressing the nuclear FKBP-anchor (H2B-2xFKBP-mCherry) together with 5C-FRB or 5C-FRB fused to TAZ fragment 290–345 (5C-FRB-290–345), the latter was slightly more nuclear even prior to stimulation. Importantly, rapamycin treatment strongly increased nuclear enrichment of 5C-FRB 290–345 compared to 5C-FRB (Fig. 3c, right panel). As a second, TAZ-specific alternative to LMB-induced nuclear entrapment, we utilized the physiological “retention factor” TEAD1. Overexpression of TEAD1 promoted 5C-TAZ nuclear accumulation in a concentration dependent manner (Supplementary Figure 4A), but had no impact on the TAZ mutant construct 5C-F52A that cannot bind TEAD^25^ or the tag alone (5C-“−“; Fig. 3d). Importantly, TEAD1 only stopped 5C-TAZ export; import remained dependent on region 290–345 as the corresponding deletion in TAZ (5C-∆290–345) diminished TEAD-triggered nuclear enrichment.

**Fig. 4:**
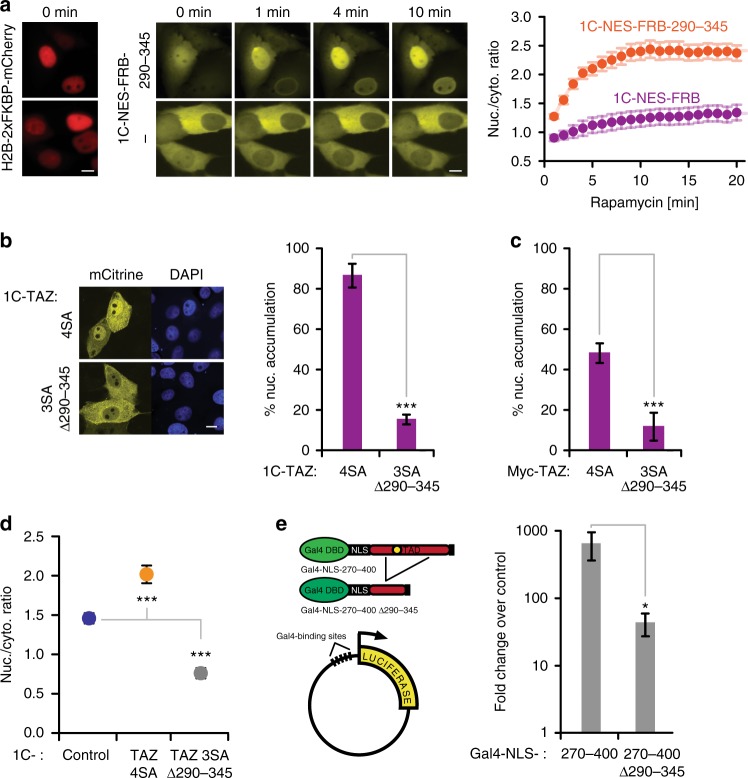
The TAZ-NLS is functionally relevant in the context of small constructs. **a** Live-cell RIS’N import assay. Fluorescence images were recorded using a Viva View system and show co-expression of H2B-2xFKBP-mCherry (red) and 1C-NES-FRB or 1C-NES-FRB-290–345 (yellow) at indicated time-points (left). Scale bar: 10 μm. Average *R*_N/C_ values ± SEM were plotted against time of rapamycin treatment. 1C-NES-FRB, *n* = 40 cells; 1C-NES-FRB-290–345, *n* = 80 cells. Differences between the two probes are significant at all time-points (*p* < 0.01, not indicated). Curves are fits to an exponential rise to maximum function. **b** Confocal fluorescence microscopy images of LLC-PK1 cells expressing 1C-TAZ constructs (4SA and 3SA ∆290–345; yellow), with DAPI stain (blue). Scale bar: 10 μm. Right: Visual quantification of cells with nuclear enrichment. Data are presented as means ± SD, *n* = 4. **c** Myc-tagged TAZ constructs (4SA and 3SA ∆290–345) were detected by immunofluorescence staining using an anti-Myc antibody and nuclear enrichment was determined by visual counting. Data are presented as means ± SD, *n* = 4. **d** Median *R*_N/C_ values of 1C-control, and indicated 1C-TAZ constructs. Data are presented as means ± SD, *n* = 3 or 6 for individual constructs. **e** Luciferase reporter system. Gal4-NLS-TAZ TAD constructs drive the expression of firefly luciferase from a plasmid with five Gal4 binding sites in the promoter region, shown on the left. Luciferase activities, normalized to the transfection control Renilla-TK, are shown as fold changes relative to the activity obtained from a Gal4-NLS-mCherry driven expression. Data are presented as means ± SD, *n* = 4. *p < 0.05, ***p < 0.001; Student’s *t*-test

We next assessed whether import, mediated by region 290–345, is relevant for smaller constructs as well and, therefore, bears physiological importance. First, we replaced the 5C-module in our RIS’N system with a single mCitrine molecule and fused it to the NES from the HIV protein Rev^45^ to increase the cytoplasmic pool of the probe under basal conditions. In live-cell experiments, rapamycin induced fast (within minutes) nuclear accumulation of the corresponding 290–345 construct (1C-NES-FRB-290–345), while the tag (1C-NES-FRB) accumulated significantly slower (Fig. 4a). Similar results were obtained using our automated-analysis pipeline on fixed cells (Supplementary Figure 4B). Second, we tested whether region 290–345 would be required for nuclear localization of full-length TAZ in the context of small tags. Deletion ∆290–345 strongly abrogated the otherwise nuclear localization of 1C-TAZ 4SA (Fig. 4b) or Myc-tagged TAZ 4SA (Fig. 4c), which demonstrated that this region was not only sufficient, but also necessary for efficient nuclear accumulation of TAZ. Moreover, automated analysis revealed nuclear exclusion of 1C-TAZ 3SA ∆290–345, in contrast to the diffusion-driven localization of 1C-control (Fig. 4d). These findings indicated that in the absence of import, mediated by region 290–345, nuclear efflux via an unknown mechanism (see below) offset the diffusion-driven nuclear localization seen with 1C-control. Thus, the functional interplay of import and export predominantly defined the steady-state cellular distribution of TAZ 4SA beyond passive diffusion.

Since region 290–345 is part of the transactivation domain (TAD; Fig. 3a), a largely disordered, acidic region essential for the expression of TAZ-target genes^46^, it was conceivable that it also contributed to gene expression, in addition to its crucial role for nuclear localization. We tested the transcriptional activity of the complete TAD (fragment 270–400) and the 290–345 deletion construct (270–400 ∆290–345) in the context of a Gal4-luciferase reporter system (Fig. 4e). A SV40-NLS ensured that all constructs were equally nuclear. Luciferase activity was ~15 fold reduced when expression was driven by the 290–345 deletion construct. In summary, we identified region 290–345 as sufficient and necessary for efficient nuclear import of TAZ in addition to its critical role for transcriptional activity.

### Region 290–345 comprises a novel NLS that mediates RAN-independent import

**Fig. 5:**
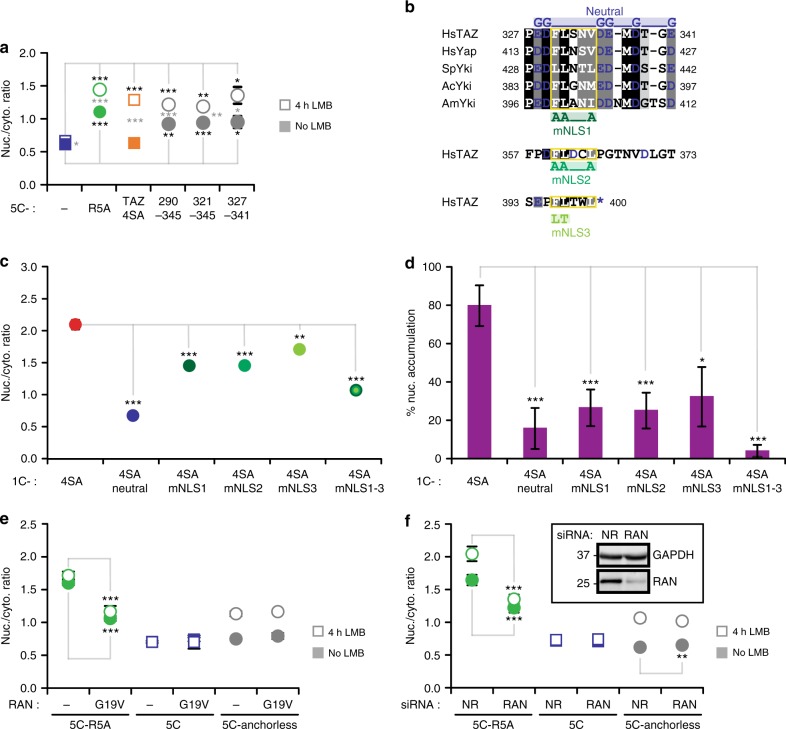
Characterization of the TAZ-NLS and its mediated import. **a** Median R_N/C_ values of control (5C), 5C-R5A, 5C-TAZ 4SA, and 5C-tagged TAZ fragments. Gray asterisks indicate statistically significant changes upon LMB treatment. Black asterisks mark significant differences relative to the control (5C), *n* = 3. **b** Sequence alignment of region 327–341 in human (Hs) TAZ with human Yap and orthologs (Yorkie, Yki) from sea slug (Ac: Aplysia californica), bee (Am: Apis mellifera), and sea urchin (Sp: Strongylocentrotus purpuratus). Positions with identical residues and conserved physicochemical properties are shown on black and dark gray background, respectively. Light gray indicates 80% conservation of properties. Negatively charged residues are depicted in blue. The central hydrophobic motif FLxx[I,V,L,M] is marked by a yellow box. Substitutions of the “neutral” mutant are shown above the alignment, mutation of the hydrophobic motif mNLS1 below. Related sequences in TAZ and mutations mNLS2 and mNLS3 are shown below. The blue star indicates the C-terminus. **c** Median R_N/C_ values of indicated 1C-TAZ constructs, *n* = 3. **d** Nuclear enrichment of indicated 1C-TAZ constructs, *n* = 3 or 6 for individual constructs. **e** Constitutively active RAN G19V inhibits classic NLS-mediated nuclear import but not that of anchorless TAZ. Median *R*_N/C_ values of 5C-R5A, the 5C-tag alone, and 5C-anchorless (TAZ 4SA F52A), when co-expressed with control  vector or RAN G19V, in the absence and presence of 4 h LMB treatment, *n* = 4. **f** siRNA-induced silencing of RAN does not affect nuclear import of anchorless TAZ. Median R_N/C_ values of 5C-R5A, the 5C-tag alone, and 5C-anchorless (TAZ 4SA F52A), when co-transfected with non-related (NR) or RAN-specific siRNA, in the absence and presence of 4h LMB treatment, *n* = 4. Insert: western blot showing the efficiency of RAN-silencing with GAPDH as loading control. An uncropped version is shown in Supplementary Figure 1B. Data are represented as means ± SD. *p < 0.05, **p < 0.01, ***p < 0.001; Student’s *t*-test

Next, we sought to map key features in region 290–345, essential for import. To narrow down the location of major import determinants, we first tested smaller fragments for their nuclear accumulation, when fused to the 5C-tag. We found that fifteen residues (327–341) retained the import capacity of region 290–345 (Fig. 5a). Strikingly, this fragment is the most acidic part of TAZ (Fig. 3a), and sequence alignment (Fig. 5b) highlighted conservation of these negative charges. Indeed, negative charges proved to be functionally essential; mutation of the six Asp and Glu residues to Gly (“neutral” mutation) reduced nuclear accumulation of 1C-TAZ 4SA to the same extent as deletion 290–345 (compare Figs 5c and 4d). We noted that the critical negative residues flanked the conserved motif FLxx(V/L/I/M) (Fig. 5b). Intriguingly, such a pattern, i.e., the hydrophobic motif flanked by negative charges, occurred at two additional regions in TAZ: at 357–373 in the TAD and in the C-terminal PDZ binding site (393–400), the latter of which has been shown to contribute to nuclear accumulation^20^. Thus, we asked if the FLxx(V/L/I/M) motif in 327–341 is critical for nuclear entry and if these other regions also confer transport capacity via this motif. Mutation of the hydrophobic residues in region 327–341 to alanine (mNLS1, Fig. 5b) significantly reduced nuclear accumulation of 1C-TAZ 4SA, as did the mutations of the other sites (mNLS2 and mNLS3, Fig. 5c, d). Albeit the effects of the individual hydrophobic mutations were subtle, simultaneous mutation of all three sites had a robust and additive effect.

Since TAZ molecules can dimerize^47,48^, we considered that a piggyback mechanism involving endogenous TAZ might contribute to the nuclear accumulation of our constructs. To address this possibility, we first downregulated endogenous TAZ using siRNA and expressed an “anchorless” TAZ mutant (5C-TAZ 4SA F52A), incapable of binding the major “retention factors” 14–3–3 and TEAD^20,21,25^. Despite efficient reduction in endogenous TAZ (Supplementary Figure 5A), the nuclear accumulation of 5C-anchlorless was not affected (Supplementary Figure 5B). As a second approach, we directly assessed the capacity of region 290–345 to bind full-length TAZ. Cells were transfected with Myc-tagged TAZ 4SA and various 1C-constructs, and immunoprecipitations were performed using GFP-trap beads. While Myc-tagged TAZ 4SA readily co-precipitated with 1C-TAZ 4SA, it showed only background association with 1C-290–345 (Supplementary Figure 5C). Conversely, deletion ∆290–345 in 1C-TAZ 4SA did not reduce the association with Myc-tagged TAZ 4SA. Therefore, the proposed TAZ-NLS in region 290–345 appears not to coincide with the major dimerization site. This conclusion is consistent with previous observations showing that dimerization is mediated by the coiled–coiled domain^48^, and some critical cysteines that reside outside of the NLS^47^. Together, these experiments ruled out a key role for endogenous TAZ in the import of the tested fragments.

Given that the identified TAZ-NLS vastly differ from the classic, positively charged NLS such as that found in SV40, we wondered whether TAZ import also depended on the small GTPase RAN. We inhibited classic import (as exemplified by 5C-R5A) by expressing the constitutively active RAN mutant G19V^49,50^ or by siRNA-mediated knockdown of endogenous RAN (Fig. 5e, f), and then tested the effect on the cellular distribution of the anchorless mutant. Neither RAN G19V expression nor RAN knockdown inhibited nuclear accumulation of 5C-tagged anchorless TAZ in the presence of LMB (Fig. 5e, f). Furthermore, nuclear localization of 1C-anchorless TAZ was also not affected by RAN G19V expression (Supplementary Figure 5D). Taken together, we identified a novel nuclear localization signal, defined by negatively charged and hydrophobic residues, that mediates import of TAZ in a RAN GTPase-independent manner. In addition to the major localization signal present in 327–341, at least two other motifs contribute to import.

### Regulation of TAZ import by RhoA activation

**Fig. 6:**
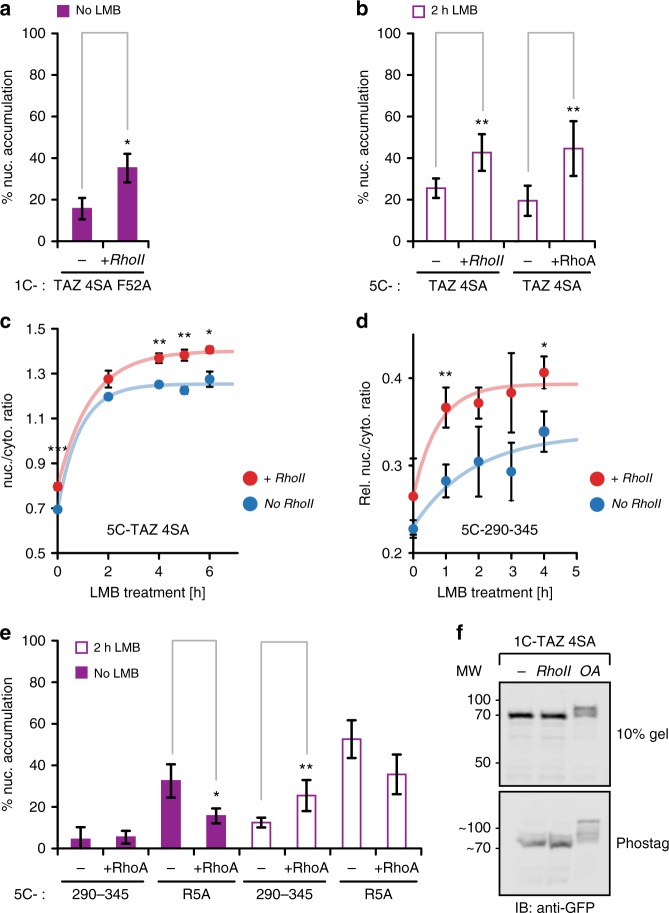
Regulation of TAZ import by RhoA. **a** Effect of the RhoA activator RhoII on the nuclear enrichment of 1C-TAZ 4SA F52A (anchorless construct), *n* = 3. **b** Nuclear enrichment of 5C-TAZ 4SA upon RhoA activation by RhoII or by co-expression of constitutively active RhoA Q63L along with 2h LMB treatment, *n* = 6. **c** RhoA activation increases LMB-induced nuclear accumulation of 5C-TAZ 4SA. LMB time course without and with RhoA stimulation, *n* = 3. **d** RhoA activation increase LMB-induced nuclear accumulation of the 5C-TAZ-NLS. LMB time course without and with RhoA stimulation, *n* = 3. Rel. nuc./cyto. ratio refers to the difference between 5C-TAZ-NLS and 5C. Curves in **c** and **d** are fits as in Fig. 3c. **e** RhoA activity selectively increases nuclear accumulation of the TAZ-NLS. 5C-290–345 and the SV40-NLS construct 5C-R5A were expressed in combination with control vector or RhoA Q63L. Following a 2 h treatment with or without LMB, nuclear accumulation was visually determined, *n* = 3, 5, or 6 for individual constructs. **f** RhoA stimulation does not induce LATS-independent TAZ phosphorylation, as detected by band shifts in western blots. Cells expressing 1C-TAZ 4SA were treated with PBS, RhoII or the phosphatase inhibitor okadaic acid (OA) for 6 h and lysates were run on conventional (upper panel) and Phos-tag (lower panel) polyacrylamide gels. After western blot, ectopic proteins were detected with anti-GFP antibody. Uncropped version shown in Supplementary Figure 6C. Data are represented as means ± SD. *p < 0.05, **p < 0.01; Student’s *t*-test

To address whether LATS-independent, cytoskeletal/RhoA-mediated regulation of TAZ might function through import control, we activated RhoA with Rho activator II (RhoII) or expressed the constitutively active mutant RhoA Q63L (Supplementary Figure 6A) and monitored TAZ localization. RhoA activity increased nuclear accumulation of the anchorless construct, 1C-TAZ 4SA F52A (Fig. 6a). It also increased the accumulation of 5C-TAZ 4SA in the presence of LMB (Fig. 6b, c), proving that the import step was enhanced. Remarkably, RhoA activation could even increase import of 5C-290–345 (Fig. 6d and Supplementary Figure 6B), and this effect was TAZ-specific, as RhoA did not enhance classic import via the SV40 R5A NLS (Fig. 6e). Since RhoA is upstream of several kinases, we tested whether its activation might have increased import through LATS-independent TAZ phosphorylation. We did not observe changes in the migration patterns of 1C-TAZ 4SA (Fig. 6f) or 1C-290–345 (Supplementary Figure 6C) on conventional polyacrylamide gels or phosphorylation-sensitive Phos-tag gels when RhoA was activated. In contrast, the phosphatase inhibitor okadaic acid (OA), which was used as a positive control, induced strong band shifts indicative of phosphorylation. Consistent with these results, elimination of potential phosphorylation sites in region 290–345 or introducing phosphomimetic mutations did not affect basic nuclear import (Supplementary Figure 7) but prevented the OA-induced band shift (Supplementary Figure 6C). In summary, our results show that facilitated import through region 290–345 was enhanced by RhoA activity. This effect was TAZ-specific but was not due to TAZ phosphorylation.

### Identification of TAZ-NES

**Fig. 7:**
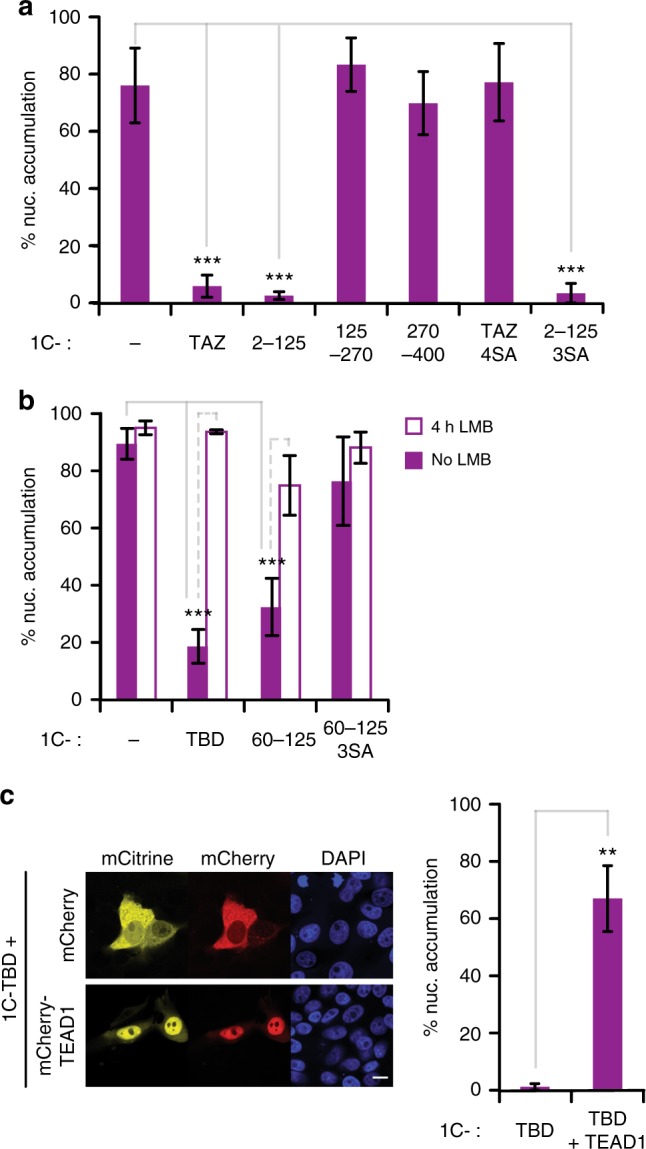
The TEAD-Binding Domain (TBD) mediates cytoplasmic accumulation. **a** Nuclear accumulation of 1C, 1C-TAZ, or 1C-tagged TAZ fragments, *n* = 4. **b** Nuclear accumulation of 1C-tagged TBD (2–60) and 14–3–3 binding region (60–125) with or without 4h LMB treatment, *n* = 4. **c** TEAD-induced nuclear accumulation of the TBD. Confocal fluorescence microscopy images of 1C-TBD (yellow), co-expressed with mCherry or mCherry-TEAD1 (red). Nuclei were visualized by DAPI staining (blue). Scale bar: 10 μm. Quantifications are shown on the right, *n* = 4. Data are represented as means ± SD. **p < 0.01, ***p < 0.001; Student’s *t*-test

Our results on the LMB-inducible nuclear accumulation of large (Fig. 2) and small TAZ constructs (Supplementary Figure 8) also suggested the presence of a CRM1-mediated export process, a view raised earlier based on the LMB sensitivity of endogenous Yap and TAZ^14,51,52^. However, the responsible NES has not been identified. As no such obvious sequence could be predicted, we followed the same fragmentation approach as shown in Fig. 3a to identify TAZ region(s) that relocated the nuclear 1C-tag to the cytoplasm (Fig. 7a). Such nuclear exclusion is a strong indicator for a functional NES. Of the three fragments tested, only 2–125 was entirely cytoplasmic, consistent with the presence of the 14–3–3 binding site in this fragment. However, 14–3–3 binding could not be the sole reason for the exclusion because mutation of the LATS-phosphorylation sites (1C-2-125 3SA) failed to promote nuclear accumulation, suggesting the presence of a NES. To verify this notion and narrow down the responsible elements, we cut the fragment into half and analyzed the distribution of the 1C-tagged subfragments (2–60, termed TEAD-binding domain (TBD) and 60–125). Both fragments revealed nuclear exclusion, but the latter re-distributed into the nucleus when the LATS phosphorylation sites were mutated (1C-60–125 3SA), in line with 14–3–3-based exclusion (Fig. 7b). LMB treatment abolished the differences between the various constructs, indicating that their initial differential distribution was not due to their inability to translocate into the nucleus. Intriguingly, when TEAD1 was co-expressed, 1C-TBD relocated to the nucleus (Fig. 7c). Thus, paradoxically, the TBD appears to have two opposite functions: it can promote cytoplasmic localization, as well as TEAD-dependent nuclear “retention” of TAZ. In the following experiments we wished to dissect the structural features responsible for these opposite functions.

### The TBD comprises a CRM1-dependent NES

**Fig. 8:**
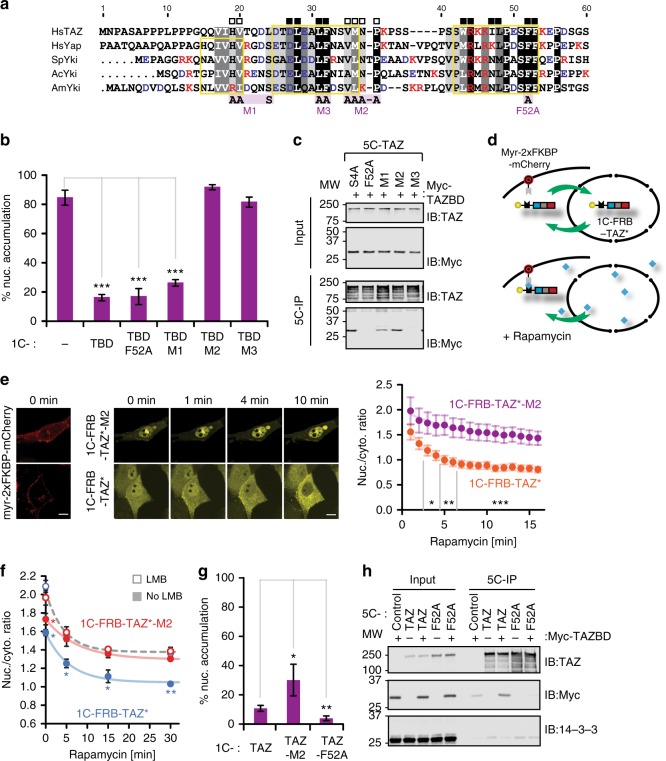
The TBD comprises a NES within the TEAD-binding site. **a** Sequence alignment of TBDs from TAZ homologs, as in Fig. 5b. Positively charged residues are depicted in red. Filled and open boxes (above alignment) highlight important or dispensable positions for TEAD binding, respectively^25^. TEAD-binding sites are boxed in yellow^54, 55^. Mutations used in this study are shown below. **b** Nuclear enrichment of 1C-TBD mutants, presented as mean ± SD, *n* = 3. **c** Impact of TBD mutations on TEAD binding. 5C-TAZ constructs and the Myc-TAZ binding domain of TEAD1 (TAZBD) were co-expressed. 5C-constructs from lysates were immunoprecipitated with GFP-trap beads. TAZ and Myc-TAZBD were detected in lysate (input) and immunoprecipitates (5C-IP) by western analysis using TAZ- and Myc-specific antibodies, respectively. **d** RIS’C model showing rapamycin-induced cytoplasmic entrapment of 1C-FRB-TAZ 4SA F52A (1C-FRB-TAZ*) by myristoylated-2xFKBP-mCherry. **e** RIS’C assay using live-cell imaging. Cells expressing Myr-2xFKBP-mCherry and 1C-FRB-TAZ* or 1C-FRB-TAZ*-M2 were imaged confocally. After the first image (t = 0 min), rapamycin was added and further images were taken. Scale bar: 10 μm. Average *R*_N/C_ values ± SEM are plotted against time. 1C-FRB-TAZ*, *n* = 43 cells, 1C-FRB-TAZ*-M2, *n* = 29 cells. Significant differences between the two probes are indicated in time-intervals. Fits represent an exponential decay to minimum function. **f** LMB sensitivity of nuclear export. Experiments and fits were performed as in (**e**), with or without additional LMB treatment (open and closed circles) for 80 min. Median *R*_N/C_ values of 1C-FRB-TAZ* (blue) and 1C-FRB-TAZ*-M2 (red) are presented as means ± SD, *n* = 3. Curves correspond to an exponential decay function. In the presence of LMB, the decays of nuclear accumulation of the constructs are indistinguishable (single fit; black dotted curve). Blue and red asterisks indicate minimum significance levels of 1C-FRB-TAZ* relative to all other conditions and 1C-FRB-TAZ*-M2 relative to LMB-treated samples, respectively. **g** Nuclear enrichment of 1C-TAZ constructs with mutations in the TBD, presented as means ± SD, *n* = 4. **h** Competition between TAZBD and 14–3–3 for TAZ binding. 5C-control and 5C-TAZ constructs were co-expressed with or without Myc-TAZBD, immunoprecipitated with GFP-trap beads, and analyzed by western blotting using TAZ-, Myc-, and 14–3–3 specific antibodies. Note weak, unspecific binding of Myc-TAZBD to 5C-control. Uncropped versions of blots are shown in Supplementary Figure 1C and 1D. *p < 0.05, **p < 0.01, ***p < 0.001; Student’s *t*-test

Sequence alignment highlighted a conserved pattern of hydrophobic residues in the TEAD-binding site vaguely reminiscent of a CRM1-dependent NES (Fig. 8a). We introduced four sets of mutations (F52A and M1-3) to probe the importance of these hydrophobic residues for nuclear exclusion of 1C-TBD. Residues mutated in F52A and M1 were dispensable, the two constructs still localized to the cytoplasm like the WT 1C-TBD (Fig. 8b). In contrast, mutants M2 and M3 accumulated in the nucleus. Further truncation analysis localized the functionally most critical region for nuclear exclusion to the first 40 residues of TAZ (Supplementary Figure 9A). Next, we assessed the TEAD-binding capacity of these mutants (vis-à-vis their localization properties) by co-immunoprecipitation experiments. To avoid complications arising from differential localizations of the mutants, we used cytoplasmic 5C-TAZ constructs and the TAZ binding domain of TEAD (TAZBD), which is evenly distributed throughout cells^53^. We found that mutations M1 and M2 maintained TEAD binding, whereas mutations M3 and F52A abrogated it (Fig. 8c), in line with previous findings^25^. Together, the results showed that mutations in M1 did not prevent TEAD binding or export. In contrast, residues mutated in M2 were essential for nuclear exclusion, F52 was critical for TEAD binding, and sites in M3 contributed to both functions. Hence, nuclear exclusion was provided primarily by a set of residues within region 2–40 that overlapped with, but was distinct from, the TEAD-binding site spanning residues 25–53 in TAZ^54,55^. These findings offered an explanation for the opposing roles of the TBD in TAZ localization. Namely, the TBD confers nuclear localization by TEAD-binding, which masks residues important for efflux. Conversely, in the absence of interaction with TEAD, the TBD can mediate efflux. To test these assumptions, we co-expressed TEAD with the various 1C-TBD constructs (Supplementary Figure 9B). TEAD expression induced nuclear accumulation of 1C-TBD, but not of the non-binding mutant F52A. When the intrinsic export function per se was corrupted, however, TEAD binding was dispensable for nuclear accumulation: binding and non-binding mutants M2 and M3 were both nuclear.

To verify that the TBD was essential for TAZ export, independent of concomitant re-entry, we employed a rapamycin-inducible sequestration in the cytoplasm (RIS’C) system, based on a cytoplasmic, membrane bound FKBP-trap and 1C-FRB-TAZ constructs comprising the “anchorless” mutations (4SA F52A) (Fig. 8d). The M2 mutations strongly diminished efflux upon rapamycin-induced cytosolic sequestration, as evidenced by the slower decay of nuclear fluorescence (Fig. 8e, Supplementary Figure 9C). Moreover, pre-treatment with LMB reduced export to background levels (Fig. 8f). These experiments verified that the TBD comprised a functional, LMB-sensitive NES. Both functions of the TBD, nuclear export and TEAD binding were also relevant for the localization of full-length TAZ. Inhibition of export by the M2 mutations (Fig. 8g) or deletion of the entire TBD (Supplementary Figure 9D) increased nuclear accumulation, whereas disruption of TEAD binding alone diminished it (F52A, Fig. 8g).

Since active RhoA increases nuclear accumulation of TAZ, we wondered if it inhibited export, in addition to stimulating import. RhoA Q63L expression had no effect on export as determined by the RIS’C system (Supplementary Figure 9E) and caused only modest increase in nuclear 1C-TBD (Supplementary Figure 9F), which was also observed with a 1C-construct comprising the Rev-NES^45^ (1C-RevNES). This suggests that RhoA activity leads to a general, weak export inhibition in addition to an increase in TAZ import.

**Fig. 9:**
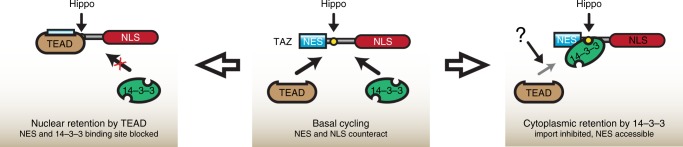
Model for NES masking- and competition-mediated regulation of nuclear export. 14–3–3 and TEAD recognition sites in the N-terminus of TAZ are in close proximity. Binding of TEAD blocks the function of the NES and might limit access of 14–3–3, thereby switching off export (left). Various regulatory inputs impeding TEAD–TAZ interaction are indicated by “question mark” (see Discussion); the NES remains accessible, leading to nuclear exit (right). The input of the Hippo pathway (on S89 phosphorylation) is indicated

Considering that 14–3–3 and TEAD bind to nearby sites in TAZ (Fig. 3a) but have opposite effects (their binding facilitates nuclear or cytosolic accumulation, respectively), we wondered if there might be a competition between these regulators. In fact, reciprocal binding of 14–3–3 and TEAD to TAZ was suggested earlier^21,56^, but those experiments did not distinguish between exclusion due to different compartmentalization of the binding partners and true competition for the TAZ binding sites. We performed co-immunoprecipitation experiments with 5C-TAZ constructs to detect binding of endogenous 14–3–3 in the cytoplasm. Co-expression of the TAZ binding domain (TAZBD) competed off 14–3–3 from 5C-TAZ, but not from 5C-TAZ F52A, which cannot bind TAZBD (Fig. 8h). These results are consistent with the higher affinity of TAZ for TEAD (in-vitro, 42 ± 19 nM^57^) compared to the lower ligand binding affinities of 14–3–3 proteins (in-vitro, ~100–500 nM^58,59^). Taken together, these latter findings extend the existing models of the regulation of TAZ distribution in two important aspects; they show that TEAD promotes TAZ nuclear “retention” by masking the newly identified TAZ-NES and suggest an intrinsic competition between TEAD and 14–3–3 for TAZ (see Fig. 9 and Discussion).

## Discussion

One of the major knowledge gaps in the rapidly growing field of Yap/TAZ biology has concerned the mechanisms and molecular requirements underlying nuclear translocation of these proteins^60^. Our studies unequivocally demonstrate that nuclear influx of TAZ is, at least in part, a mediated process, i.e., it is not only brought about by passive diffusion of “free” cytosolic TAZ, but also involves a cellular machinery with the capacity to translocate even large proteins (>150 kD) into the nucleus. The responsible NLS, which is both necessary and sufficient for efficient mediated transport is “unusual” in that it contains negatively charged residues, flanking the hydrophobic motifs FLXXV/L/I/M. This signature is present in all Yap/TAZ homologs except Drosophila Yorkie which, instead, comprises a functionally unrelated NLS^61^. Moreover, this motif is also contained in a small set of other proteins, the majority of which are nuclear and involved in transcription (Supplementary Figure 10). Interestingly, the NLS is also required for the transcriptional activity of TAZ per se, beyond its localization function (Fig. 4e). Together these findings afford two propositions: the identified motif in TAZ could be the prototype of a new class of bona fide NLS, and these sequences may have double function, supporting nuclear entry and transactivation. The TAZ-NLS is of medium strength, i.e., it is considerably weaker than the supraphysiological SV40-NLS, which drives exclusive nuclear localization. This propensity allows fine-tuned, graded regulation of nuclear TAZ accumulation. Keeping with this notion, we have identified three copies of this NLS motif in TAZ. While we have shown that the first (327–341) is critical for efficient import, the others (357–373 and 393–400) may also contribute (see Fig. 2c for LMB-induced import of a construct lacking the critical region 290–345). Of interest, the most C-terminal one of these, coinciding with the PDZ binding site, has been demonstrated to facilitate nuclear accumulation of TAZ in a previous study^20^, raising the interesting possibility that this motif can simultaneously partake in import and “retention”.

Further, consistent with its “non-canonical” nature, the TAZ-NLS conducts import independent of RAN. This raises the intriguing possibility that, given its biochemical properties, it could directly interact with the hydrophobic, positively charged Phe/Gly (FG) meshwork of the NPC, similar to a small set of other transcription factors (e.g., NXF family, and possibly β-catenin)^62,63^. Alternatively, the motif may mediate TAZ binding to transporters that provide RAN-independent entry. Characterization of this novel import mechanism warrants further studies. However, it has to be emphasized that the overall nuclear accumulation of TAZ depends not only on its import and export, but also on the distributions of its “retention factors” and modifying kinases (e.g., LATS), which are likely affected by importins and RAN^30,53^.

We also provide evidence that TAZ import itself is a regulated process, and that the NLS is sufficient to confer this regulation. We found that RhoA activation increased nuclear influx of TAZ and the isolated NLS, but not of cargos with classic NLS. How can active RhoA promote TAZ-specific entry? A recent elegant study^29^ has shown that mechanical loading of the cytoskeleton leads to nuclear flattening, which in turn deforms the nuclear pore, thereby increasing its permeability for molecules with low mechanical stability, such as Yap. Our finding that RhoA stimulates TAZ import is in agreement with the general conclusion that the cytoskeleton modulates nuclear entry. However, mechanically induced rise in pore permeability, a priori, is not expected to be sequence-specific, while our observations indicated TAZ-specific transport. It is plausible, however, that the force-induced changes in the NPC may also stimulate sequence-specific, mediated import of TAZ in addition to passive influx. Alternatively, RhoA could increase influx by modulating TAZ and/or the import machinery. While the NLS and its vicinity contain several phosphorylation sites, we did not detect RhoA-induced TAZ phosphorylation, and our mutagenesis studies suggested that these phosphorylation sites were not required for basal import. However, these findings do not exclude the possibility that CDK1 or Src-mediated phosphorylation of TAZ can impact TAZ import^64,65^.

It is worthwhile to consider the interplay between the classic, cytoplasmic “retention mechanism”, and influx regulation. We found that the Hippo pathway mitigates the mediated TAZ influx into the nucleus. Mechanistically, 14–3–3 binding increases the molecular weight of TAZ by ~58 kD which is more than the size difference between nuclear 1C-TAZ 4SA and cytoplasmic 3C-TAZ 4SA. Thus, a simple increase in the complex size could be sufficient to reduce influx and thereby “retain” TAZ in the cytoplasm. In fact, this interpretation seems to be consistent with findings published while our work was under review^32^. While that paper considers export as the main regulated step of Yap localization (see below), the authors also found that a LATS-independent mutant (5SA) exhibited a significantly higher import rate than WT in cancer-associated fibroblasts (CAFs), although the change was the opposite in normal fibroblasts. In this regard our epithelial cells show CAF-like behavior. In addition, we cannot exclude that 14–3–3 controls the TAZ-NLS, as described for other proteins^66,67^.

While LMB-induced nuclear accumulation of TAZ has been reported^14,52^, it was unclear whether this phenomenon was primarily due to nuclear entrapment of TAZ-interacting proteins or reflected the inhibition of TAZ export per se via its own NES. In this study we have identified the responsible TAZ-NES, which is also conserved in Yap. Therefore, the emerging role of export in Yap regulation^32^ further increases the importance of this finding. The identified TAZ-NES overlaps with the binding site for TEAD, consistent with earlier studies showing that TEAD binding is a rate-limiting factor in TAZ efflux^25^. This observation puts TEAD-dependent regulation of TAZ traffic in a new light: TAZ nuclear “retention” entails TEAD-mediated masking of the NES.

We also found that TEAD can compete off 14–3–3 from TAZ. It is tempting to speculate that the inverse scenario might also exist, namely that 14–3–3 could compete off TEAD from TAZ under certain conditions. If this is so, it would enhance efflux according to our masking model. In fact, our masking model, as well as the idea of reciprocal competition, align well with recent findings showing that a LATS phosphorylation mutant Yap (which cannot bind 14–3–3) exhibits diminished export, while a TEAD-binding-deficient mutant has a higher export rate than WT in CAFs^32^. Given the emerging role of posttranslational modifications (phosphorylation, palmitoylation, glutathionylation) of both TEAD and TAZ^68–71^, which can alter their affinity for each other, along with the increasing list of TEAD-interacting proteins that could control Yap/TAZ binding^68,72^, such regulation of efflux might signify a major mechanism in TAZ (and Yap) trafficking. Along these lines, a recent study found that osmotic stress induces the nuclear efflux of both TEAD and TAZ, and also reduces the association of these proteins^30^. This finding is also consistent with the potential liberation of the TAZ-NES via TEAD debinding. Nonetheless, Yap/TAZ efflux might be brought about by alternative mechanisms as well, such as the recently described Merlin-mediated nuclear export^73^. Further studies are warranted to characterize the various modes of TAZ export, the corresponding (pathophysiological) stimuli, and the underlying changes in the interaction of Yap/TAZ with their binding partners.

In summary, localization of TAZ (and Yap) has been suggested to be affected by a multitude of (potentially non-exclusive) mechanisms, the relative contribution of which remains to be determined in a stimulus- and cell type-specific manner. These entail (a) LATS-dependent and (b) LATS-independent release from cytoplasmic binding partners (14–3-3^20,21^ and AMOT^26–28^, respectively), which might augment the most diffusible pool of TAZ; (c) increase in the permeability of the nuclear pore;^29^ (d) stimulation of NLS-mediated import (current study); and (e) a modulation of export^32^, at least in part by masking the NES (current study).

Taken together, we propose a refined model for TAZ shuttling (Figure 9), which includes three novel aspects: the presence of an NLS, the potency of which is enhanced by RhoA; the presence of an NES, which is masked by TEAD binding; and a competition between 14–3–3 and TEAD. Identification of the molecular determinants of influx and efflux should facilitate pharmacological targeting of Yap/TAZ shuttling and function, which play central roles in cell growth, differentiation, carcinogenesis, and fibrosis.

## Methods

### Reagents and antibodies

For western blot analysis, proteins were detected using the following antibodies: anti-TAZ (BD Biosciences, 560235, 1:1000), anti-RAN (Cell Signaling Technology, #4462, 1:1000), anti-c-Myc (Santa Cruz Biotechnology, SC-40, 1:1000), anti-pan-14–3–3 (Santa Cruz Biotechnology, SC-629, 1:1000), and anti-GFP (SC-8334, 1:1000). Jackson ImmunoResearch Laboratories was the source for HRP, IRDye680RD, and IRDye800CW conjugated secondary antibodies. Alexa 488 or 555 conjugated secondary antibodies were from Invitrogen. HRP conjugated secondary antibodies were used in 1:5000 for western blot analysis, fluorescent secondary antibodies 1:10000. Leptomycin B, Rapamycin, and okadaic acid were purchased from Sigma Aldrich and RhoII activator from Cytoskeleton Inc. Phos-tag gels (Wako Pure Chemical Industries, Ltd.) were ordered from Cedarlane.

### Cell culture

LLC-PK1 cells^74^ were cultured in low glucose DMEM, supplemented with 10% FBS and penicillin/streptomycin (all Life Technologies). For live-cell confocal imaging, cells were incubated in synthetic medium (20 mM HEPES, pH 7.4, 130 mM NaCl, 3 mM KCl, 1 mM CaCl_2_, 1 mM MgCl_2_, 5 mM glucose) in the absence of serum at room temperature. In all experiments where indicated, cells were treated with RhoII (1 μg/ml) for 6h and/or LMB (20 ng/ml) and/or Rapamycin (1 μM) or the corresponding vehicles for the indicated times.

### Expression plasmids and siRNA transfection

5xGal4-TATA-luciferase was a gift from Richard Maurer^75^, pPGS-3HA-TEAD1 and pCMX-Gal4-TEAD2 were gifts from Kunliang Guan^55,76^ (Addgene plasmids #46756, #33055, and #33107). HA-tagged RAN WT and G19V constructs were a gift from Andrew Wilde^50^. The Myc-tag expression vector with Asc1 and Pac1 sites was a gift from Gerald Gish. TAZ 4SA was a gift from Jeff Wrana^18^. Other plasmids used as template in polymerase chain reactions (PCR) were gifts form Tony Pawson. All fluorescently tagged constructs were based on pcDNA3.1(−), with mCitrine or mCherry coding regions inserted using Xho1 and EcoR1 sites. Additional mCitrine copies were successively introduced using the EcoR1 and BamH1 sites in the pcDNA3.1(−)-mCitrine constructs and Mfe1 and BamH1 sites flanking the mCitrine encoding insert. The ligation of Mfe1 and EcoR1 cleaved ends destroyed these restriction sites. An internal EcoR1 site close to the 3′end of the mCitrine insert allowed further insertion-rounds. TAZ, MRTF, RhoA, and mCherry coding sequences were inserted using Asc1 and Pac1 sites. Point mutations, deletions and insertions of NLS, NES, FRB domains, FKBP domains, the Lyn-kinase  myristoylation site, H2B, or linker regions were created by standard overlapping PCR techniques. For luciferase experiments, the TEAD2 coding region in pCMX-Gal4-TEAD2 was replaced by a DNA fragment encoding the SV40-NLS and TAZ fragment 270–400 using EcoR1 and Kpn1 sites. Asc1 and Pac1 sites flanking the TAZ region were used to exchange it with TAZ 270–400 ∆290–345 or the coding region of mCherry. For Myc-TEAD expression constructs, fragments encoding aminoacids 17–426 (“full-length” with the N-terminus of isoform 2) or 208–426 (“TAZBD”) were cloned into the Myc-tag vector via Asc1 and Pac1 sites. For all PCR reactions a high fidelity proof reading polymerase (Phusion; Thermo Scientific) was used. Restriction enzymes were purchased from New England BioLabs. All constructs generated were verified by sequencing. Transfections were performed using X-tremeGENE 9 (Roche Applied Science) or jetPRIME (PolyPlus Transfection SA) according to the manufacturer’s instructions. Controls for samples coexpressing TEAD or RhoA constructs comprised empty vector. Porcine-specific siRNAs used in knockdown experiments were directed against the following sequences : TAZ 5′-CAAGAACATACACCTACGGTTGT-3′;^7^ RAN 5′-GCAACAAAGTGGATATTAA-3′. Oligonucleotides were synthesized and purchased from Thermo Scientific or Sigma-Aldrich. NR control siRNA was obtained from Applied Biosystems. Cells were transfected with 100 nM siRNAs alone or together with plasmids using jetPRIME and were analysed for silencing or the cellular localization of co-transfected constructs 48h later.

### Luciferase reporter assay

Cells were transfected with 5xGal4-TATA-luciferase reporter, comprising 5 Gal4 binding sites and either pCMX-Gal4-NLS-mCherry, pCMX-Gal4-NLS-TAZ 270–400, or pCMX-Gal4-NLS-TAZ 270–400 ∆290–345. Furthermore, the normalizing plasmid pRL-TK (Promega) and pcDNA3-mCherry as carrier DNA were added to the transfection mix. Renilla luciferase and firefly luciferase activities in cell lysates were measured using a reporter assay system (Dual Luciferase; Promega) in a luminometer (Lumat 9507; Berthold). Firefly/renilla ratios are expressed as fold changes compared to the firefly/renilla ratio of the control^74^.

### Immunoprecipitation and western blotting

To examine interactions between endogenous 14–3–3 and/or indicated, transiently expressed, constructs, LLC-PK1 cells were harvested from 10 cm dishes and lysed in Tris-buffered saline, comprising 1 mM EDTA, 20 mM Sodium Fluoride, and 1% Triton X-100 and supplemented with 1 mM PMSF, 1 mM Sodium Vanadate, and Complete Mini Protease Inhibitor (Roche). Lysates were spun at 4 °C, 12,000 rpm for 5 min to remove cell debris and analyzed for protein content (BCA Protein Assay; Pierce Biotechnology). Supernatants were incubated with GFP-trap beads (ChromoTek) for 1 h and beads were washed three times afterwards. Bound proteins were eluted using SDS-PAGE sample buffer and subjected to SDS-PAGE followed by western blot analysis using nitrocellulose membranes. Aliquots of each input were run in parallel to monitor expression levels. Samples for Phos-tag gels were prepared by lysing cells directly in 2x SDS-PAGE sample buffer. Phos-tag gels were run and proteins transferred onto membranes according to the manufacturer’s instructions. Immunodetections were either performed using ECL Plus reagents (GE Healthcare Life Sciences) and a GS800 densitometer or the infrared Odyssey imager (LI-COR).

### Immunofluorescence and fluorescence confocal microscopy

For immunofluorescence microscopy, LLC-PK1 cells were grown on glass coverslips, transfected with Myc-tagged constructs and fixed with 4% paraformaldehyde. Following permeabilization with 0.1% Triton X-100 and blocking with BSA, cells were first incubated with anti-c-Myc (1:100) primary antibody and then with fluorescently conjugated secondary antibody (Alexa 488 or 555, 1:1000). For fluorescence microscopy, cells were transfected with constructs indicated in the figures and fixed with 4% paraformaldehyde. DAPI (Lonza) was used to counterstain nuclei. Coverslips were mounted on slides using fluorescent mounting medium (Dako). Images were captured using a WaveFX spinning-disk microscopy system (Quorum Technologies) equipped with ORCA-Flash4.0 digital camera, driven by the MetaMorph software. Visual quantification of the cellular localization was performed by scoring cells as “cytoplasmic” (cyto.) if nuclear fluorescence was lower than cytoplasmic fluorescence, as “pan” if both compartments had similar fluorescence levels, or as “nuclear” (nuc.) if nuclear fluorescence was higher. For simplicity, in all figures, except Supplementary Figure 3B, only nuclear fractions are reported. At least 10 randomly selected fields per condition were analysed, with a total of at least 100 scored cells. A minimum of three independent experiments were conducted. All image processing was done according to the Journal’s guidelines.

### Automated localization analysis

LLC-PK1 cells were plated into multi-well cell-culture plates (Falcon) and co-transfected with constructs indicated in the figures. After 24 h, cells were fixed with 4% paraformaldehyde and nuclei were stained with DAPI (Lonza). A total of 25 images per well were acquired using the ImageXpress Micro system (Molecular Devices) and analysed with the inbuilt Multi Wavelength Translocation analysis module. Nuclear-to-cytoplasmic ratios were calculated and the median of each distribution was reported.

### Live-cell experiments

For export studies, LLC-PK1 cells plated into 35-mm glass bottom dishes (MaTek) were co-transfected with Myr-Lyn-2xFKBP-mCherry and either 1C-FRB-TAZ 4SA F52A or 1C-FRB-TAZ 4SA F52A-M2 constructs. Experiments were performed 24 h later. Cells were preincubated in synthetic medium at room temperature for at least 15 min. Using a WaveFX spinning-disk microscopy system, images were acquired every minute, before and for at least 15 min after rapamycin addition. For import studies, LLC-PK1 cells on 35-mm glass bottom dishes were co-transfected with H2B-2xFKBP-mCherry and either 1C-FRB-NES-290–345 or 1C-FRB-NES constructs. After 24 h, images were acquired every 30 sec and time-averaged to one minute, before and for at least 15 min after rapamycin addition using a Viva View system (Olympus). Data were collected from at least 29 cells for each condition from nuclear and cytoplasmic regions within the same cell and a control region outside of cells for background correction. Data points were excluded if the cytoplasmic mean fluorescence intensities were less than 20% above background. Images were analysed using ImageJ^77^.

### Statistics

For each construct, typically more than 100 transfected cells were visually inspected per experiment. For automated analysis, 100–5000 transfected cells were analysed per construct and experiment. N identifies the number of repeats of independent experiments. All experiments were repeated at least three times. Data are presented as bar diagrams (visual analysis, luciferase experiments, and database searches) or scatter plots (automated analysis and live-cell experiments) as mean ± standard deviation (SD) or standard error of the mean (SEM). Statistical significance was determined by one-sided ANOVA, if more than two groups existed. All ANOVA analyses resulted in *P* < 0.001. Unpaired, two-tailed Student’s *t* tests were performed and *P* < 0.05 was accepted as significant. Statistical analysis was done in Excel (Microsoft). Significance values: ns, not significant (*P* > 0.05), *P < 0.05, **P < 0.01, ***P < 0.001. For curve fitting, SigmaPlot (Systat Software, Inc.) and Prism (GraphPad Software, Inc.) were used.

### Reporting Summary

Further information on research design is available in the Nature Research Reporting Summary linked to this article.

## Electronic supplementary material

Supplementary Information

Reporting Summary

## Data Availability

The datasets generated during and/or analysed during the current study are available from the corresponding author on reasonable request.
