# Supplementary material for: Mediated nuclear import and export of TAZ and the underlying molecular requirements
Source: Nat Commun. 2018 Nov 23;9:4966. doi: 10.1038/s41467-018-07450-0 (PMC6251892; doi:10.1038/s41467-018-07450-0)
Supplement: Supplementary file 1 — Supplementary Information [file 41467_2018_7450_MOESM1_ESM.pdf]

SUPPLEMNTARY INFORMATION – Mediated nuclear import and export of TAZ and the underlying molecular requirements

Supplementary Figure 1

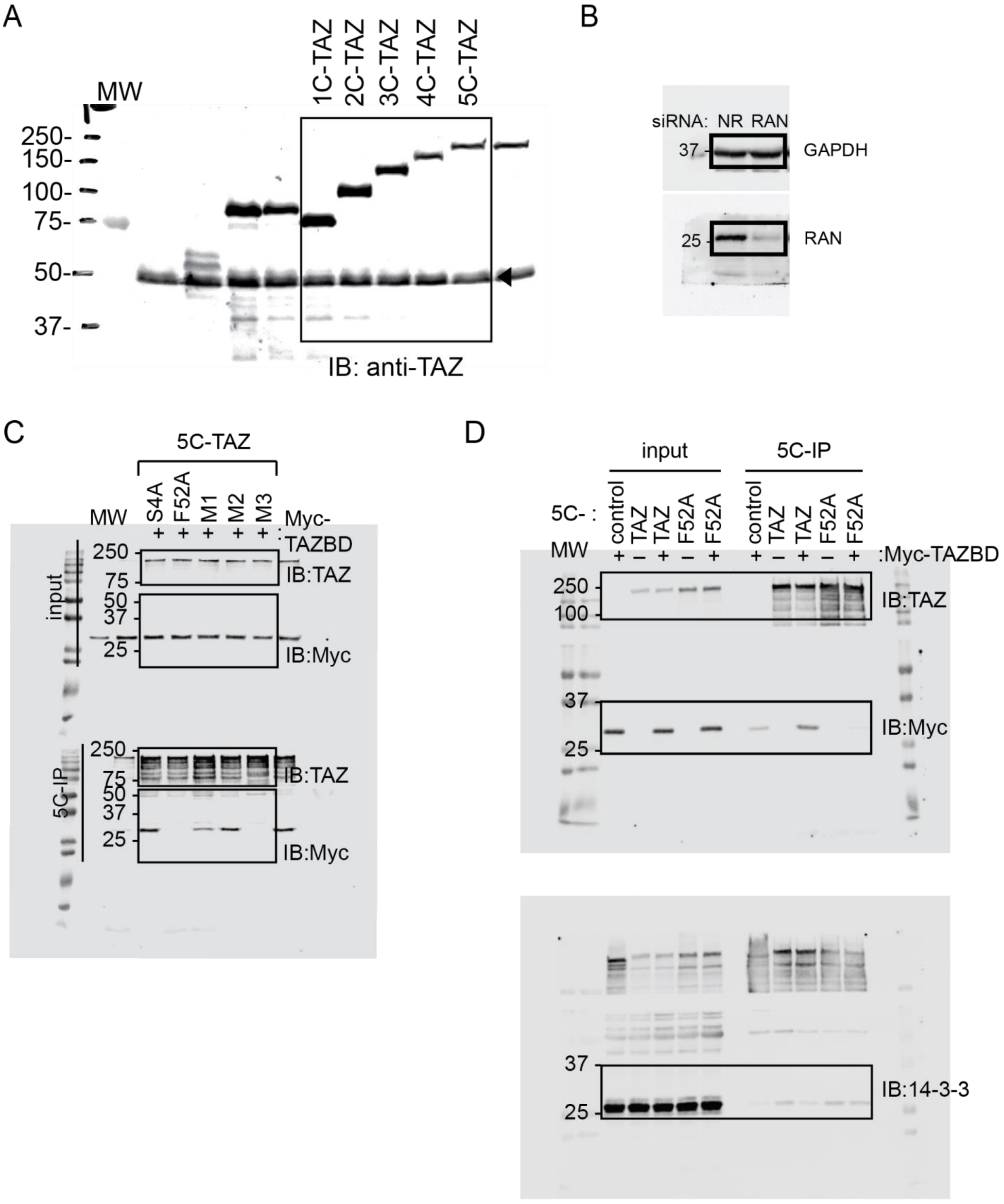

Supplementary Figure 1 – Uncropped western blots.

A: Molecular weight of TAZ fused to the different mCitrine-tags, corresponding to the western blot shown in Figure 1B.

B: siRNA-induced silencing of endogenous RAN. GAPDH serves as loading control. Corresponds to the blot shown in the insert of Figure 5F.

C: Effect of TBD mutations on TEAD binding. Corresponds to the western blot shown in Figure 8C.

D: Competition between TEAD and 14-3-3 for TAZ binding. Corresponds to the western blot shown in Figure 8H.

Supplementary Figure 2

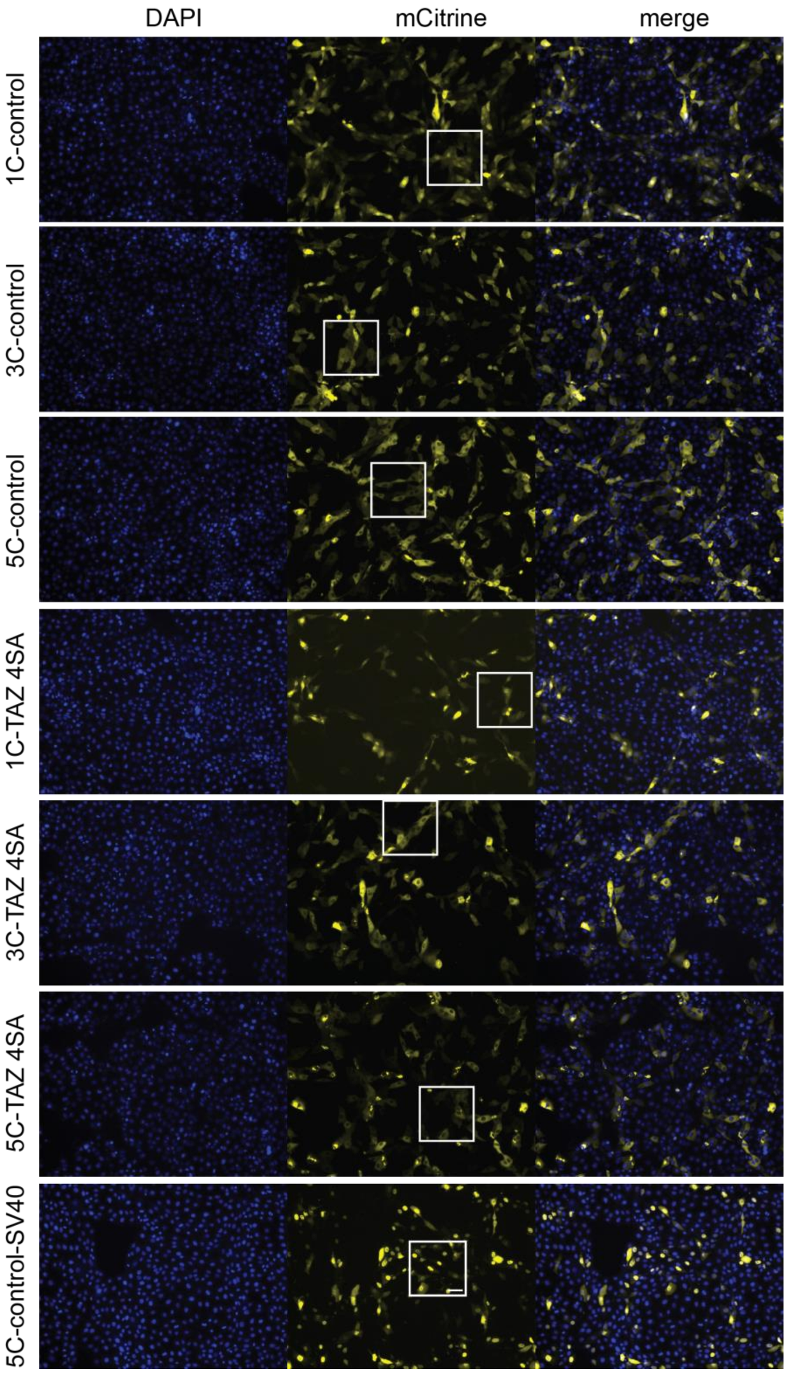

Supplementary Figure 2 – Cellular distribution of designed TAZ constructs in LLC-PK1 cells.

Fluorescence microscopy images of cells expressing the indicated constructs, using the ImageXpress Micro system. Images depict mCitrine fluorescence (yellow), DAPI (blue), and a merge of both channels. White boxes refer to regions shown in Figure 1C. Scale bars represent 50 µm.

Supplementary Figure 3

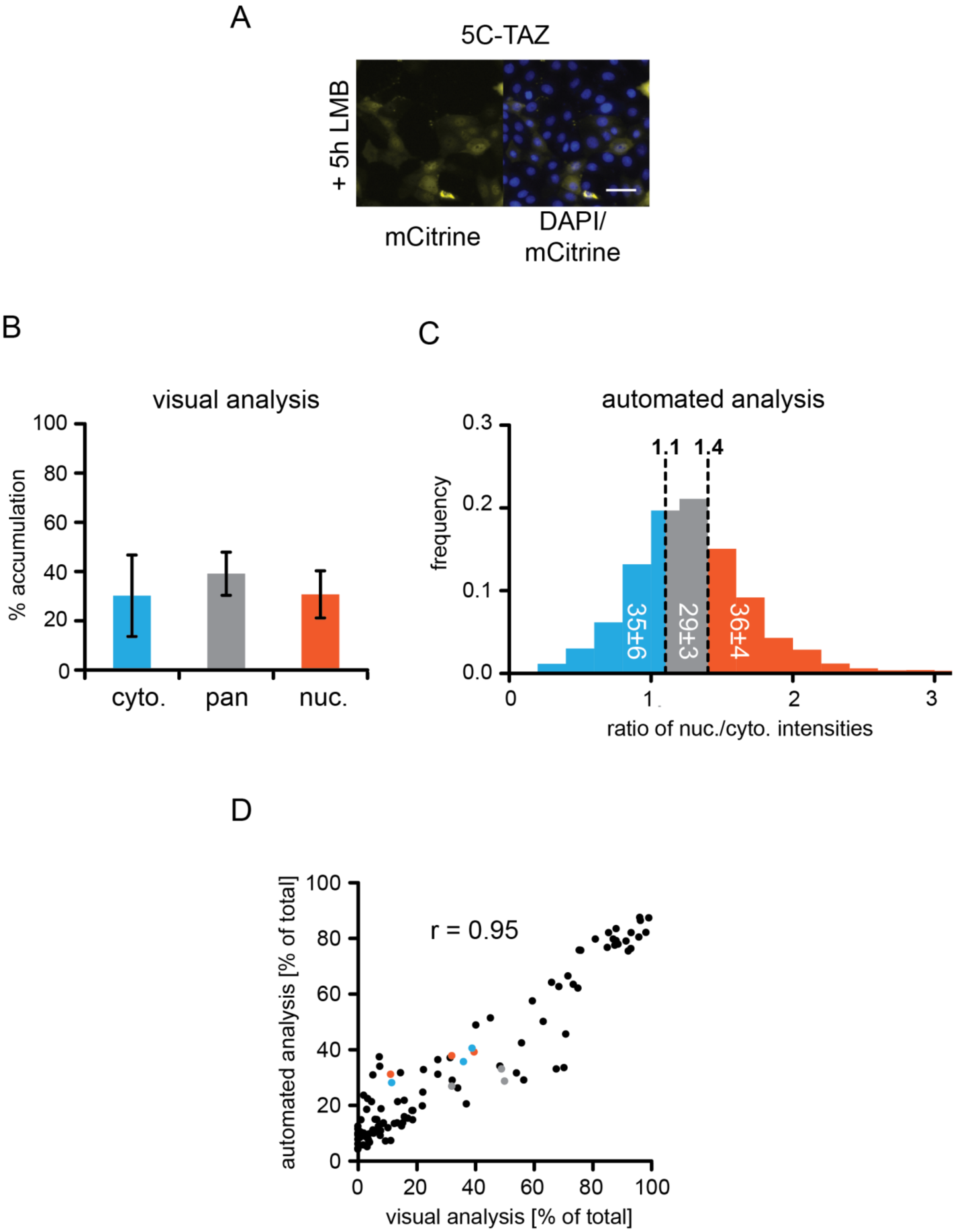

Supplementary Figure 3 – Correlation between visual and automated quantification methods.

A: Fluorescence microscopy images of cells expressing 5C-TAZ, after 5h LMB treatment. Images as described in Supplementary Figure 2.

B: Visual quantification of the intracellular distribution of 5C-TAZ after 5h LMB treatment, as shown in A. Percentages of cells, showing predominantly cytoplasmic (cyto.) or nuclear (nuc.) fluorescence or an even distribution (pan) of fluorescence are indicated. A minimum of 100 cells in total for each construct were analyzed, n=3. Data are presented as means ± SD.

C: Automated analysis of the same samples as in B. Cytoplasmic, pan, and nuclear localizations (determined by visual categorization in B) correspond to the colour-matched portions of the distribution and can be defined by the general threshold ratios 1.1 and 1.4 as boundaries. Percentages of the total population are shown.

D: Comparison of visual and automated analysis methods. Cytoplasmic, pan and nuclear localizations, as determined by visual categorization and the related portions of the corresponding automated measurements were plotted against each other. For the comparison of both analysis methods, we measured the localization of 5C-control, 5C-control-SV40, 5C-TAZ, 5C-TAZ 4SA, and 5C-TAZ 290-345 without and with 5h LMB treatment in triplicate. Individual data points for 5C-TAZ after 5h LMB treatment are shown in blue (cytoplasmic fraction), grey (pan fraction) and red (nuclear fraction) and correspond to the measurements summarized in B and C. The Pearson's correlation coefficient of 0.95 attested high consistency between the two analysis methods.

Supplementary Figure 4

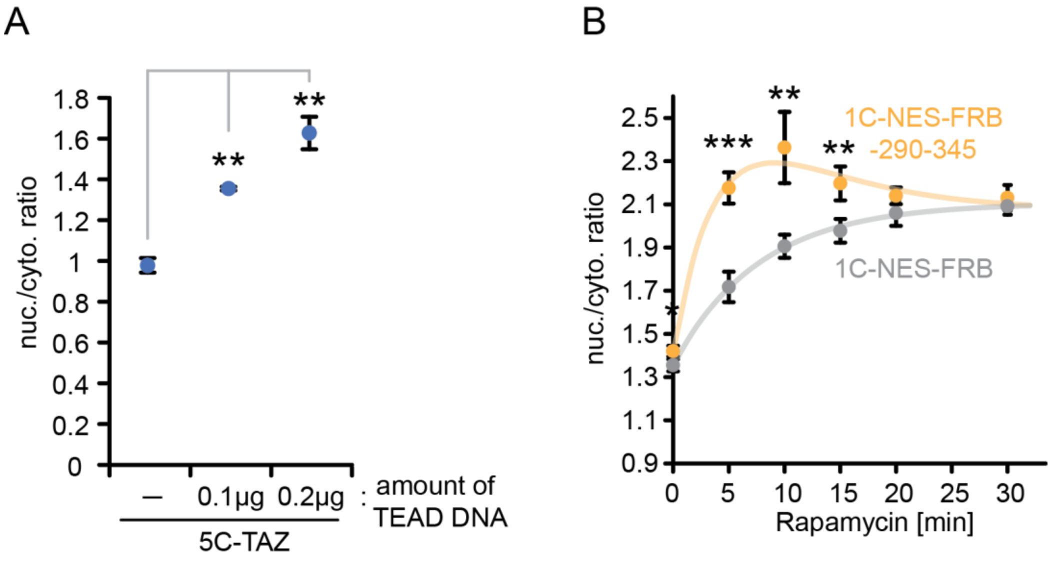

Supplementary Figure 4 – TEAD over-expression driven nuclear accumulation of 5C-TAZ and demonstration of the nuclear import capacity of region 290-345.

A: Concentration-dependent nuclear recruitment of TAZ by TEAD. Median $R_{N/C}$ values of 5C-TAZ, coexpressed with mCherry or increasing amounts of mCherry-TEAD1, n=3.

B: Data from the modified RIS'N system described in Figure 4A. Median $R_{N/C}$ values were determined after cell fixation, n=4. Curves correspond to double exponential fits.

Data are represented as means ± SD. **p<0.01, ***p<0.001; Student's t-test.

Supplementary Figure 5

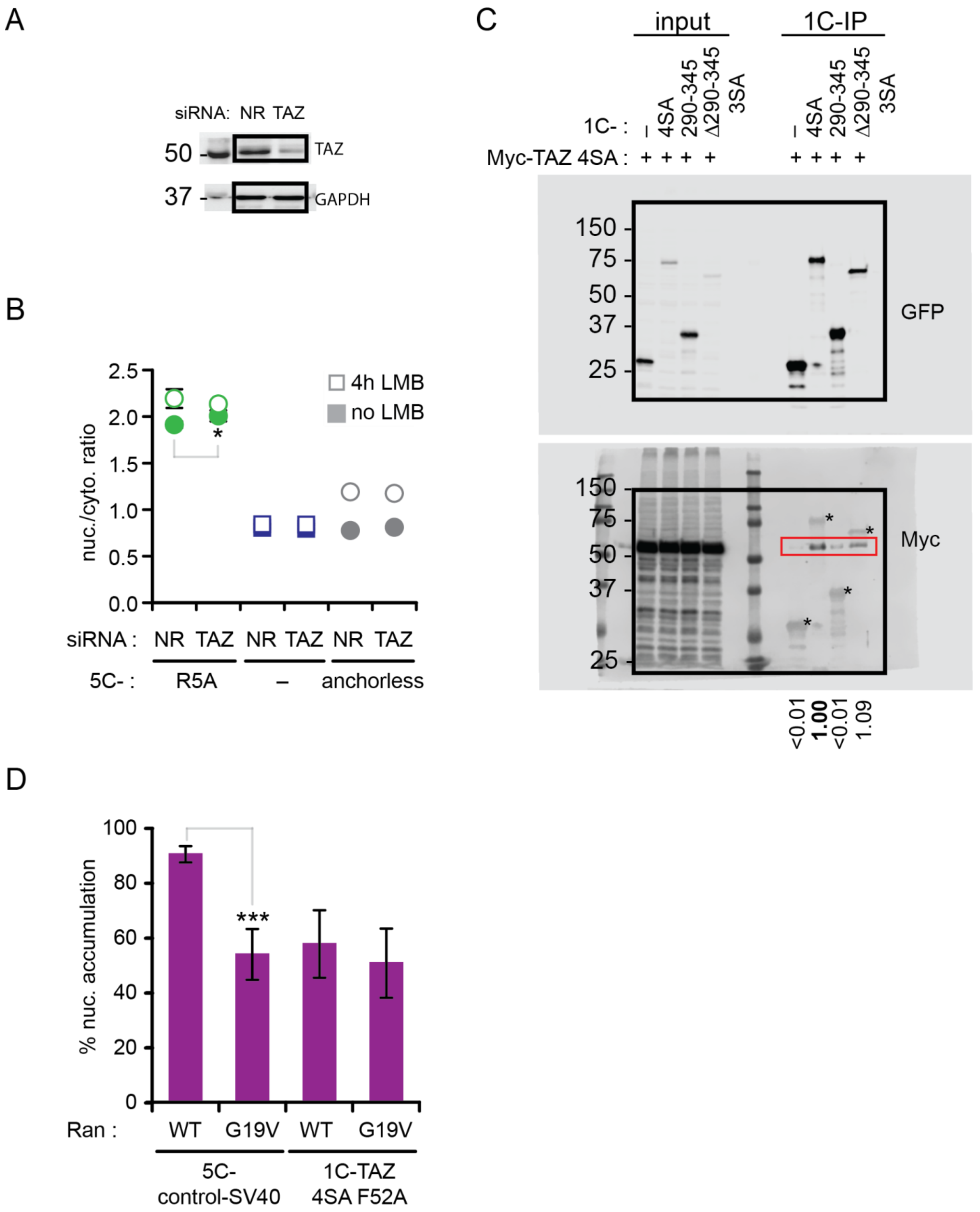

Supplementary Figure 5 – Characterization of TAZ nuclear import: role of TAZ dimerization and the GTPase RAN.

A: Western-blot showing siRNA-induced silencing of endogenous TAZ. GAPDH serves as loading control.

B: siRNA-induced TAZ silencing has no effects on the cellular distribution of anchorless TAZ. Median $R_{N/C}$ values of 5C-R5A, the 5C-tag alone, and 5C-anchorless (TAZ 4SA F52A) upon co-transfection with non-related (NR) or porcine TAZ-specific siRNA are shown, in the absence and presence of 4h LMB treatment, n=4.

C: Binding of 1C-constructs to Myc-TAZ 4SA, assessed by coimmunoprecipitation. 1C, 1C-TAZ 4SA, 1C-290-345 and the deletion construct 1C-TAZ Δ(290-345) 3SA were co-expressed with Myc-TAZ 4SA and immunoprecipitated with GFP-trap beads. Proteins in the lysate and in the precipitate were detected by western blot analysis using GFP- and Myc-specific primary and fluorescently labeled secondary antibodies. In the 1C-IP lanes, bleed-through signals from the GFP-channel are marked by asterisks and the specific Myc-signals are highlighted by a red box. Quantifications of the Myc-bands in the immunoprecipitates were normalized to the amount of expressed and immunoprecipitated 1C-constructs.

D: RAN-independent import of 1C-anchorless TAZ. Nuclear enrichment of 5C-control-SV40 and 1C-TAZ 4SA F52A, when coexpressed with WT RAN or the constitutive active mutant RAN G19V, which inhibits classic NLS-mediated import, n=6 or 13 for individual constructs.

Data are represented as means ± SD. *p<0.05, ***p<0.001; Student's t-test.

Supplementary Figure 6

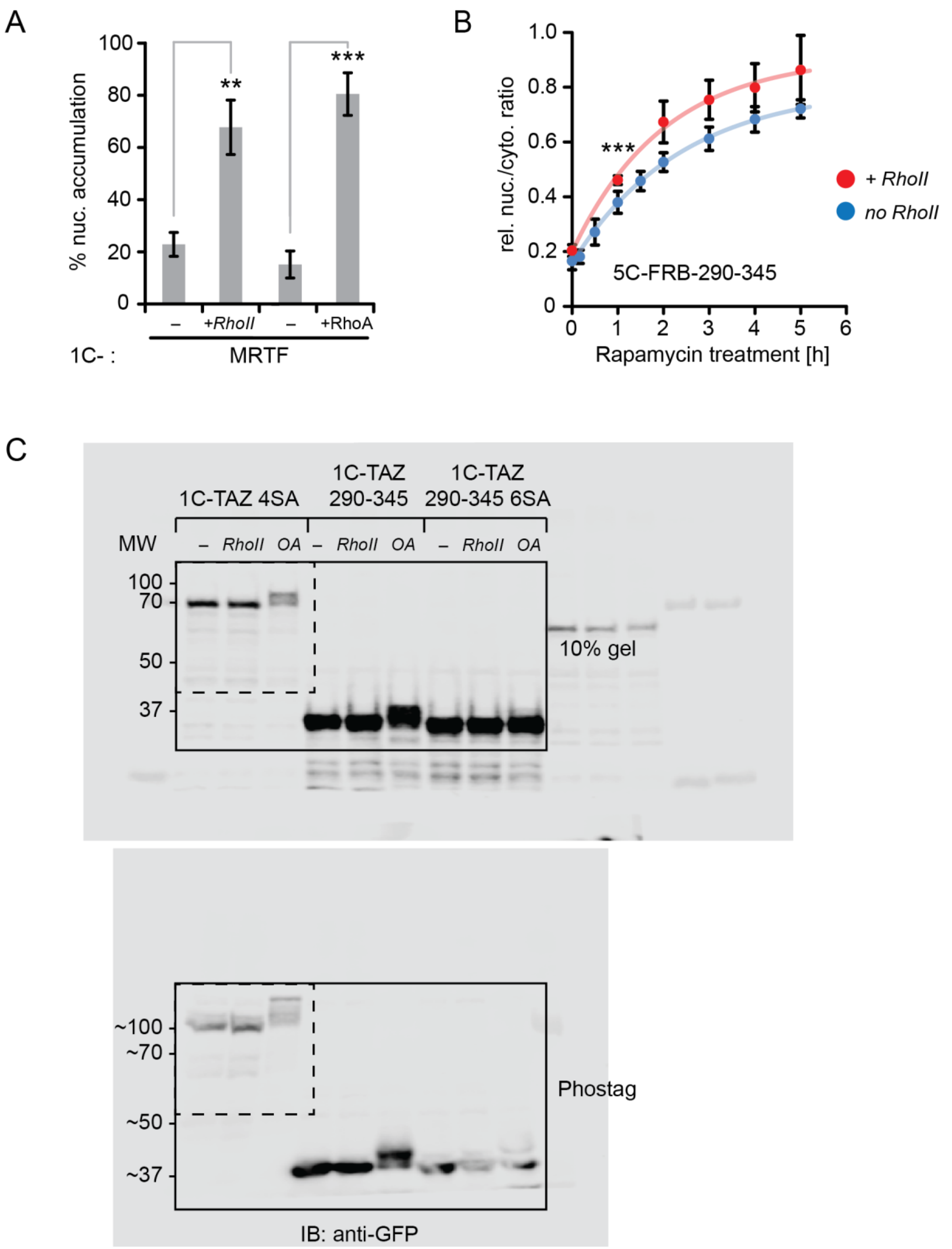

Supplementary Figure 6 – The impact of Rho activation on TAZ nuclear import and phosphorylation.

A: Functionally relevant Rho activation by RhoII addition or RhoA Q63L expression was verified by the ensuing nuclear accumulation of MRTF, which is highly responsive to RhoA-induced changes in the actin cytoskeleton, n=3 and 6 for individual constructs.

B: RhoII stimulates rapamycin-induced nuclear accumulation of 5C-FRB-290-345. Relative $R_{N/C}$ values for 5C-FRB-290-345 were calculated by subtracting the median $R_{N/C}$ values of the control 5C-FRB from those of 5C-FRB-290-345, n=3, 4, 6, or 10 for various conditions.

C: Uncropped western blots corresponding to the western blots depicted in Figure 6F (dotted boxes). Additional samples 1C-TAZ 290-345 and 1C-TAZ 290-345 6SA are shown. The upper and lower western blots were obtained using conventional and Phos-tag polyacrylamide gels, respectively. Membranes were probed with anti-GFP antibody.

Data are represented as means ± SD. **p<0.01, ***p<0.001; Student's t-test.

Supplementary Figure 7

A

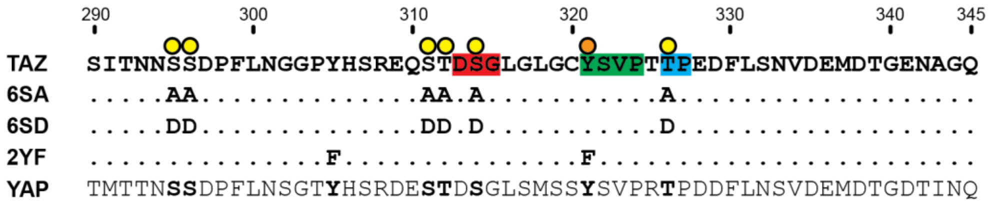

B

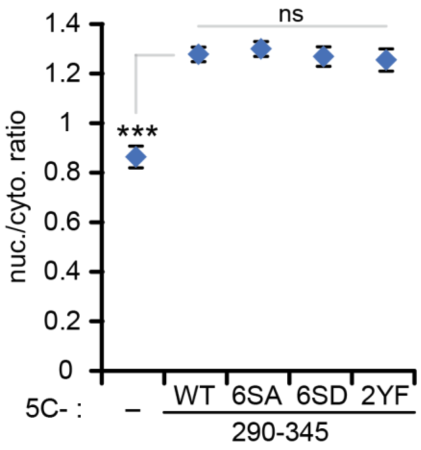

Supplementary Figure 7 – Phosphorylation sites in region 290-345 and their impact on import. A: Conserved phosphorylation sites and applied mutations in the TAZ-NLS. Region 290-345 in human TAZ and the homologues sequence in human YAP are shown at the top and bottom, respectively. Mutated residues in constructs 6SA, 6SD, and 2YF are indicated. Yellow and orange circles highlight serine/threonine and tyrosine phosphorylation sites conserved in both molecules[1], respectively. The SCF-phosphodegron is shown on red background [2], a potential Abl SH2 binding site on green background [3], and a potential WW binding site on blue background [4].

B: Phosphorylation sites and phosphorylation-dependent binding motifs in the TAZ-NLS are irrelevant for its basic import function. Median $R_{N/C}$ values of 5C, 5C-290-345 and mutant constructs, when incubated with LMB for 2h. Data are presented as means ± SD, n=4.

n/s: no significant difference, ***p<0.001; Student's t-test.

Supplementary Figure 8

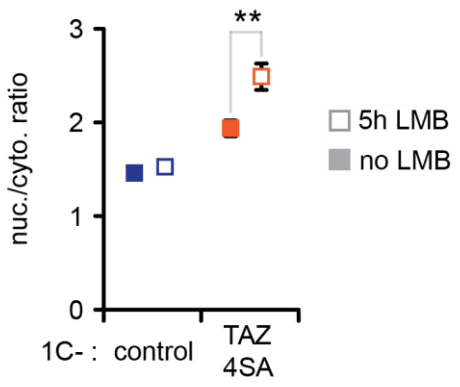

Supplementary Figure 8 – LMB induces nuclear accumulation of 1C-TAZ 4SA.

Automated localization analysis of 1C constructs with and without 5h LMB treatment. LMB increases nuclear localization of 1C-TAZ 4SA, but not 1C-control. This suggests that the cellular distribution of 1C-control does not depend on LMB-sensitive export. Median $R_{N/C}$ values are presented as means ± SD, n=3. **p<0.01; Student's t-test.

Supplementary Figure 9

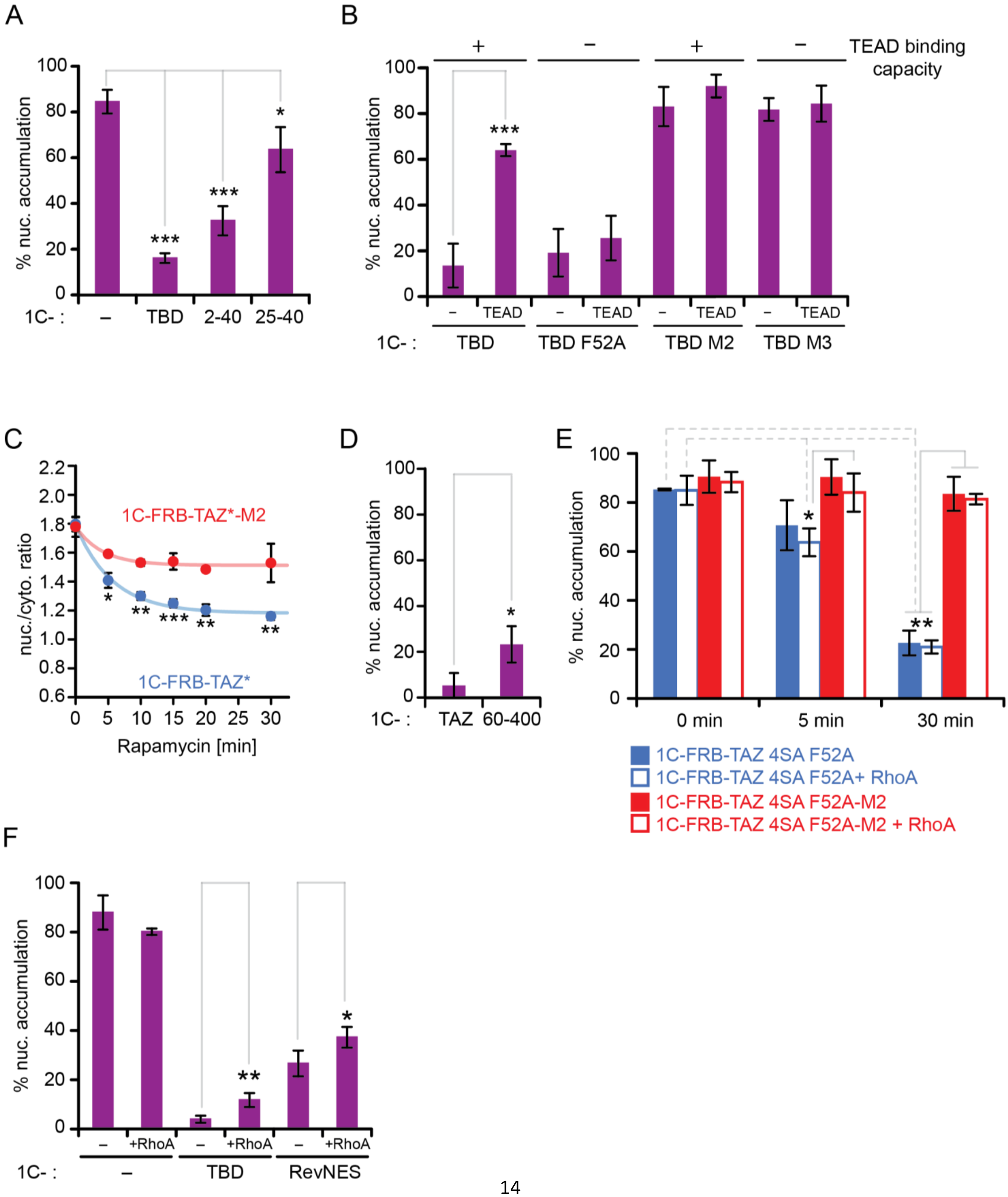

Supplementary Figure 9 – Characterization of the molecular underpinning of the TBD-mediated export and the effect of TEAD-binding and RhoA activation.

A: Mapping of the NES within the TBD. Nuclear enrichment of the 1C-constructs, n=3.

B: Induced nuclear accumulation of the TBD depends on TEAD binding. Expression mCherry-TEAD1 (TEAD) induces nuclear accumulation of 1C-TBD. Mutation F52A abolishes TEAD binding and prevents TEAD-induced relocation. TBD mutants M2 and M3 accumulate in the nucleus and hence are not responsive to TEAD expression, n=4.

C: RIS'C assay on fixed cells. Median $R_{N/C}$ values of 1C-FRB-TAZ* and 1C-FRB-TAZ*-M2, plotted against time of rapamycin treatment, n=3. Data was fit to an exponential-decay-to-minimum function using SigmaPlot, Systat Software, Inc. TAZ*: stands for the anchorless mutation TAZ 4SA F52A.

D: Deletion of the TBD (1C-TAZ 60-400) increased nuclear accumulation of TAZ, n=4.

E: RIS'C assay, testing the effect of constitutive active RhoA Q63L on export. 1C-FRB-TAZ 4SA F52A quickly accumulated in the cytoplasm upon rapamycin treatment, unlike the M2 mutant construct. RhoA Q63L coexpression (RhoA) did not affect export of either construct, n=3.

F: RhoA activation increased the nuclear accumulation of the TBD and a construct comprising the NES of HIV Rev. Nuclear accumulation of 1C, 1C-TBD, and 1C-RevNES in the absence or presence of coexpressed RhoA Q63L (RhoA), n=4.

Data are represented as means ± SD. *p<0.05, **p<0.01, ***p<0.001; Student's t-test.

Supplementary Figure 10

A

GeneName	Hit	Function	Localisation	classic NLS
ANK2	eyessvsEDFLSSVDEEnkadeak			
AP2A1	slgptpeEAFLSELEPPAPEspmalla			
C17orf53	eeefedeDFLSAVEDAEnrftgsl		nuclear	yes
CIC	etesdhdDAFLSIMSPEiqlplpp	transcription	nuclear	yes
CREBZF	geetedmDFLSGLELADlldprqp	transcription	nuclear	no
CYP2A7	nspqdfiDSFLIHMQEEEknpntef			
DPF2	krilepdDFLDDLDDEDYEedtpkrr	transcription	nuclear	yes
EXO5	fsdlsdsEFLEFLDLEDaqeskal	DNA binding	nuclear	no
FAM160A1	dgdspdpEMFLQSLTEEgsvssac			
GTF2A1L	trdadenEFLGNIDGGDlkvpeee	transcription	nuclear	yes
MTERF3	nissfdsELFLEELDElpplspm	transcription (mit.)	nuclear	no
NMD3	dderqyqDFLEDLEEDEairknvn	ribosomal component	nuclear	yes
PDZRN4	wledernEFLEELNLEMLEeehneam			
PIPSL	ydvmqdpEFLQSVLEnlpgvdp			
PPARA	dlesplsEEFLQEMGNIQEisqsige	transcription	nuclear	no
PSMD4	ydvmqdpEFLQSVLEnlpgvdp	proteasome component	nuclear	yes
SLC28A1	glenmgaDFLESLEEgqlprsd			
TIPIN	dvtstelDPFLTNLSESEmfasels	DNA replication	nuclear	yes
TNRC18	fleeassDQFLPSLEDplagmna	transcription	nuclear	yes
WWTR1	ysvpttpEDFLSNVDEMDtgenagq	transcription	nuclear	no
YAP1	ysvprtpDDFLNSVDEMDtgdtinq	transcription	nuclear	no
ZNF316	dheeeddEDFLAEVAEEENEppglwsa	transcription	nuclear	yes
ZXDA	qpsgggdDFFLVLLDPVGGDvetagsg	transcription	nuclear	yes
ZXDB	ggggggdDFFLVLLDPVGGDvetagsg	transcription	nuclear	yes
ZXDC	aeddsdgDSFLVLLEvphggaa	transcription	nuclear	no

B

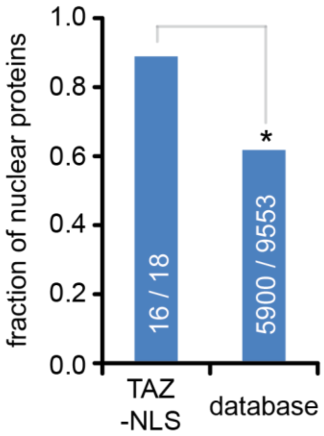

Supplementary Figure 10 – Occurrence of TAZ-NLS-like sequences in other proteins.

A: Prediction of proteins bearing TAZ-NLS like sequences. The TAZ-NLS consensus motif [ED][^RK]{0,1}FL[^RK][^RK][ILMV][^RK]{0,5}[ED] was deduced from sequence alignment. Details concerning the motif description can be found here: http://elm.eu.org/infos/help.html, under regular expressions[5]. SLiMsearch4[6] with default disorder setting and 7 flanking residues was used to search the consensus motif in the human proteome. Matching sequences were further selected for a maximum net charge of -5 and surface availability larger 50%. These hits and the corresponding gene names are listed. Functions for nuclear proteins were deduced from uniprot[7]. Localization information was used from the Human Protein Atlas available from www.proteinatlas.org[8]. The localization data available from v18.proteinatlas.org can be found here: https://www.proteinatlas.org/download/proteinatlas.tsv.zip. Grey background indicates that the localization was undefined, uncertain, or the protein was secreted or it was a transmembrane protein. Classic NLS were predicted using cNLS Mapper[9]. TAZ and Yap gene names are on blue background. Mit.: mitochondrial

B: Proteins bearing TAZ-NLS-like sequences are more often nuclear than proteins in the proteinatlas database. For the comparison, we included only those proteins from our search in A which had a defined intracellular localization (non-grey background). Correspondingly, proteins with undefined or uncertain localization, secreted proteins and transporter/ion channel were excluded from the database.

*p<0.05, calculated assuming a binomial distribution for a random data set.

REFERENCES

1 Hornbeck, P. V. *et al.* PhosphoSitePlus, 2014: mutations, PTMs and recalibrations. *Nucleic Acids Res* **43**, D512-520, doi:10.1093/nar/gku1267 (2015).

2 Liu, C. Y. *et al.* The hippo tumor pathway promotes TAZ degradation by phosphorylating a phosphodegron and recruiting the SCF{beta}-TrCP E3 ligase. *J Biol Chem* **285**, 37159-37169, doi:10.1074/jbc.M110.152942 (2010).

3 Songyang, Z. *et al.* SH2 domains recognize specific phosphopeptide sequences. *Cell* **72**, 767-778 (1993).

4 Verdecia, M. A., Bowman, M. E., Lu, K. P., Hunter, T. & Noel, J. P. Structural basis for phosphoserine-proline recognition by group IV WW domains. *Nat Struct Biol* **7**, 639-643, doi:10.1038/77929 (2000).

5 Dinkel, H. *et al.* ELM 2016--data update and new functionality of the eukaryotic linear motif resource. *Nucleic Acids Res* **44**, D294-300, doi:10.1093/nar/gkv1291 (2016).

6 Krystkowiak, I. & Davey, N. E. SLiMSearch: a framework for proteome-wide discovery and annotation of functional modules in intrinsically disordered regions. *Nucleic Acids Res* **45**, W464-W469, doi:10.1093/nar/gkx238 (2017).

7 Apweiler, R. *et al.* UniProt: the Universal Protein knowledgebase. *Nucleic Acids Res* **32**, D115-119, doi:10.1093/nar/gkh131 (2004).

8 Thul, P. J. *et al.* A subcellular map of the human proteome. *Science* **356**, doi:10.1126/science.aal3321 (2017).

9 Kosugi, S., Hasebe, M., Tomita, M. & Yanagawa, H. Systematic identification of cell cycle-dependent yeast nucleocytoplasmic shuttling proteins by prediction of composite motifs. *Proc Natl Acad Sci U S A* **106**, 10171-10176, doi:10.1073/pnas.0900604106 (2009).
