# Supplementary material for: Mediated nuclear import and export of TAZ and the underlying molecular requirements
Source: Nat Commun. 2018 Nov 23;9:4966. doi: 10.1038/s41467-018-07450-0 (PMC6251892; doi:10.1038/s41467-018-07450-0)
Supplement: Supplementary file 2 — Reporting Summary [file 41467_2018_7450_MOESM2_ESM.pdf]

nature research

Corresponding author(s): Andras Kapus, Michael Kofler

# Life Sciences Reporting Summary

Nature Research wishes to improve the reproducibility of the work that we publish. This form is intended for publication with all accepted life science papers and provides structure for consistency and transparency in reporting. Every life science submission will use this form; some list items might not apply to an individual manuscript, but all fields must be completed for clarity.

For further information on the points included in this form, see Reporting Life Sciences Research. For further information on Nature Research policies, including our data availability policy, see Authors & Referees and the Editorial Policy Checklist.

Please do not complete any field with "not applicable" or n/a. Refer to the help text for what text to use if an item is not relevant to your study. For final submission: please carefully check your responses for accuracy; you will not be able to make changes later.

## Experimental design

1. Sample size

Describe how sample size was determined.

A minimum of three independent experiments were done for each condition. In each experiment at least 100 cells or large cell populations were evaluated. This is in line with standard cell biology protocols.

2. Data exclusions

Describe any data exclusions.

In live imaging experiments, transfected cells showing cytoplasmic fluorescence less that 20% above background were omitted since this very low level of fluorescence precludes correct data interpretation.

3. Replication

Describe the measures taken to verify the reproducibility of the experimental findings.

Each experiment was repeated as indicated in the manuscript. These are true biological and not technical replicates.

4. Randomization

Describe how samples/organisms/participants were allocated into experimental groups.

We used the same cell line throughout the work.

5. Blinding

Describe whether the investigators were blinded to group allocation during data collection and/or analysis.

Unbiased automated methods were used to determine construct distributions. Careful quantitative analysis was used to measure and prove good correlation between visually and automatically determined results.

Note: all in vivo studies must report how sample size was determined and whether blinding and randomization were used.

6. Statistical parameters

For all figures and tables that use statistical methods, confirm that the following items are present in relevant figure legends (or in the Methods section if additional space is needed).

| n/a | Confirmed | |
|---|---|---|
| ☐ | ☒ | The exact sample size ($n$) for each experimental group/condition, given as a discrete number and unit of measurement (animals, litters, cultures, etc.) |
| ☐ | ☒ | A description of how samples were collected, noting whether measurements were taken from distinct samples or whether the same sample was measured repeatedly |
| ☐ | ☒ | A statement indicating how many times each experiment was replicated |
| ☐ | ☒ | The statistical test(s) used and whether they are one- or two-sided<br>*Only common tests should be described solely by name; describe more complex techniques in the Methods section.* |
| ☒ | ☐ | A description of any assumptions or corrections, such as an adjustment for multiple comparisons |
| ☐ | ☒ | Test values indicating whether an effect is present<br>*Provide confidence intervals or give results of significance tests (e.g. P values) as exact values whenever appropriate and with effect sizes noted.* |
| ☐ | ☒ | A clear description of statistics including central tendency (e.g. median, mean) and variation (e.g. standard deviation, interquartile range) |
| ☐ | ☒ | Clearly defined error bars in all relevant figure captions (with explicit mention of central tendency and variation) |

*See the web collection on statistics for biologists for further resources and guidance.*

## Software

Policy information about availability of computer code

7. Software

| | |
|---|---|
| Describe the software used to analyze the data in this study. | Excel, SigmaPlot, ImageJ, ImageXpress, Metamorph |

For manuscripts utilizing custom algorithms or software that are central to the paper but not yet described in the published literature, software must be made available to editors and reviewers upon request. We strongly encourage code deposition in a community repository (e.g. GitHub). *Nature Methods* guidance for providing algorithms and software for publication provides further information on this topic.

## Materials and reagents

Policy information about availability of materials

8. Materials availability

| | |
|---|---|
| Indicate whether there are restrictions on availability of unique materials or if these materials are only available for distribution by a third party. | No |

9. Antibodies

| | |
|---|---|
| Describe the antibodies used and how they were validated for use in the system under study (i.e. assay and species). | Antibodies used were: anti-TAZ (BD Biosciences, 560235), anti-c-Myc (Santa Cruz Biotechnology, SC-40), anti-pan-14-3-3 (Santa Cruz Biotechnology, SC-629), and anti-GFP (SC-8334). Anti-Myc and anti-GFP are against tags so their verification was straightforward comparing lysates from cells with/without transfection. Anti-TAZ visualized the bands with the expected molecular weight shift of various tagged constructs. The Anti-14-3-3 antibody (reported to be used in >200 citations) gave one single sharp band at the expected molecular weight and it also specifically visualized tagged versions. Anti-Ran (Cell Signaling, #4462) visualized a single band, which was diminished by a Ran-specific siRNA. |

10. Eukaryotic cell lines

| | |
|---|---|
| a. State the source of each eukaryotic cell line used. | LLC-PK1 cells were used, as in many of our previous studies (>20). The subclone of these cells (clone 4) used in this work was a line provided by R.C. Harris, Vanderbilt University School of Medicine, Nashville, TN, and was extensively used in our previous study. |
| b. Describe the method of cell line authentication used. | These are authenticated and immortalized classic porcine proximal tubule cell line, available at ATCC. |
| c. Report whether the cell lines were tested for mycoplasma contamination. | Yes, they were mycoplasma free. |
| d. If any of the cell lines used are listed in the database of commonly misidentified cell lines maintained by ICLAC, provide a scientific rationale for their use. | No commonly misidentified cell line was used. |

## Animals and human research participants

Policy information about studies involving animals; when reporting animal research, follow the ARRIVE guidelines

11. Description of research animals

| | |
|---|---|
| Provide all relevant details on animals and/or animal-derived materials used in the study. | No animals were used in this study. |

Policy information about studies involving human research participants

12. Description of human research participants

| | |
|---|---|
| Describe the covariate-relevant population characteristics of the human research participants. | There were no human research participants in this study. |
